# Multiscale model of primary motor cortex circuits predicts *in vivo* cell-type-specific, behavioral state-dependent dynamics

**DOI:** 10.1016/j.celrep.2023.112574

**Published:** 2023-06-09

**Authors:** Salvador Dura-Bernal, Samuel A. Neymotin, Benjamin A. Suter, Joshua Dacre, Joao V.S. Moreira, Eugenio Urdapilleta, Julia Schiemann, Ian Duguid, Gordon M.G. Shepherd, William W. Lytton

**Affiliations:** 1 Department of Physiology and Pharmacology, State University of New York (SUNY) Downstate Health Sciences University, Brooklyn, NY, USA; 2 Center for Biomedical Imaging and Neuromodulation, Nathan S. Kline Institute for Psychiatric Research, Orangeburg, NY, USA; 3 Department of Physiology, Northwestern University, Evanston, IL, USA; 4 Centre for Discovery Brain Sciences, Edinburgh Medical School: Biomedical Sciences, University of Edinburgh, Edinburgh, UK; 5 Center for Integrative Physiology and Molecular Medicine, Saarland University, Saarbrücken, Germany; 6 Aligning Science Across Parkinson’s (ASAP) Collaborative Research Network, Chevy Chase, MD, USA; 7 Department of Neurology, Kings County Hospital Center, Brooklyn, NY, USA; 8 Department of Psychiatry, Grossman School of Medicine, New York University (NYU), New York, NY, USA; 9 Present address: Department of Biomedical Engineering, University of Basel, Allschwil, Switzerland; 10 Lead contact

## Abstract

Understanding cortical function requires studying multiple scales: molecular, cellular, circuit, and behavioral. We develop a multiscale, biophysically detailed model of mouse primary motor cortex (M1) with over 10,000 neurons and 30 million synapses. Neuron types, densities, spatial distributions, morphologies, biophysics, connectivity, and dendritic synapse locations are constrained by experimental data. The model includes long-range inputs from seven thalamic and cortical regions and noradrenergic inputs. Connectivity depends on cell class and cortical depth at sublaminar resolution. The model accurately predicts *in vivo* layer- and cell-type-specific responses (firing rates and LFP) associated with behavioral states (quiet wakefulness and movement) and experimental manipulations (noradrenaline receptor blockade and thalamus inactivation). We generate mechanistic hypotheses underlying the observed activity and analyzed low-dimensional population latent dynamics. This quantitative theoretical framework can be used to integrate and interpret M1 experimental data and sheds light on the cell-type-specific multiscale dynamics associated with several experimental conditions and behaviors.

## In brief

Dura-Bernal et al. develop a realistic computational model of the motor cortex circuitry, incorporating physiological and anatomical data across different cell types. Their model successfully predicts *in vivo* neural responses for different cell types, behaviors, and manipulations and generates testable predictions about the underlying biophysical cellular and circuit mechanisms.

## Graphical Abstract

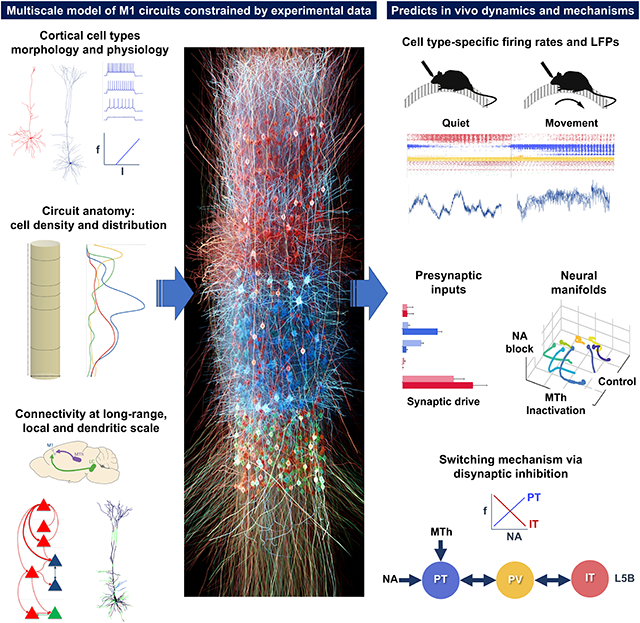

## INTRODUCTION

Understanding cortical function requires studying its components and interactions at different scales: molecular, cellular, circuit, system, and behavior. Biophysically detailed modeling provides a tool to integrate, organize, and interpret experimental data at multiple scales and translate isolated knowledge into an understanding of brain function.

Modern anatomical, physiological, and genetic techniques allow an unprecedented level of detail to be brought to the analysis and understanding of cortical microcircuits.^1,2^ In particular, several neuron classes can now be identified based on distinct gene expression, morphology, physiology, and connectivity. Cortical excitatory neurons are broadly classified by their axonal projection patterns into intratelencephalic (IT), pyramidal tract (PT), and corticothalamic (CT) types.^3-5^ Recent research has revealed that connections are specific to cell type and location and can vary even at different depths within the same cortical layer.^6-8^

The primary motor cortex (M1) plays a central role in motor control. We and others have extensively studied mouse M1 circuits experimentally and characterized cell subclasses and many cell-type- and sublaminar-specific local and long-range connections.^9-12^ A major focus of these anatomical and physiological studies has been the distinct cell classes of layer 5 (L5): L5B PT cells, the source of the corticospinal tract and other PT projections, and L5 IT cells, which project bilaterally to cortex and striatum. Morphology and physiology differ across the two types. L5 IT cells are thin tufted and show spike frequency adaptation. L5B PT cells are thick tufted and show little spike frequency adaptation but strong sag potentials. Their *in vivo* spiking dynamics have also been shown to differ.^13^ In terms of synaptic interconnectivity, these two cell types exhibit a strong asymmetry: connections go from IT to PT cells but not in the opposite direction.^8,14^ The strength of local excitatory input connections to PT cells also depends on their position within L5B, with PT cells in the upper sublayer receiving the strongest input from L2/3.^6,15-17^ These and several other highly specific local and long-range wiring patterns are likely to have profound consequences for our understanding of cortical dynamics, information processing, function, and behavior.^18^

A critical unanswered question in the motor system and, more generally, in neural systems^19-21^ is how cell and circuit dynamics relate to behavior. Both IT and PT cell types play a role in motor planning and execution and have been implicated in motor-related diseases.^22^ We have previously shown that the hyperpolarization-activated current (*I*_h_), a target of noradrenergic neuromodulation, is highly expressed in PT cells and affects its synaptic integration and electrophysiological properties.^23,24^ *In vivo* studies also reveal that noradrenergic neuromodulatory input from the locus coeruleus (LC) and long-range inputs from thalamus and cortex causally influence M1 activity and behavioral states.^25-27^ Specifically, blocking noradrenergic input to M1 impaired motor coordination,^26^ and disrupting the cerebellar-recipient motor thalamic projections to M1 can impair dexterity^27^ or block movement initiation.^28^ These modulatory and long-range projections target specific cell types and have been characterized via *ex vivo* slice experiments.^23,29,30^ However, how these inputs alter the dynamics of different M1 neural populations during motor behavior, and the exact cellular and circuit mechanisms underpinning these changes, remains largely unknown. A biologically realistic model of M1 can address this knowledge gap by generating hypotheses and predictions that relate circuit dynamics to function and behavior.

Previous models of M1 circuits are scarce and lack the detail across scales required to address these questions adequately. The M1 models developed by Morita and Kawaguchi^31^ and Hoshino et al.^32^ only included a single layer with two cell types. Heinzle et al.^33^ proposed a microcircuit model of the frontal eye field with four layers and multiple cell types. However, these circuit models included highly simplified neuron models with limited biophysical detail and no morphological detail. Our previous work modeling M1^34,35^ incorporated neuron models with 5-compartment morphologies and multiple ionic channels, as well as several cell types distributed over five cortical layers and connected based on layer and cell type. However, these models did not include tuning to cell-type-specific electrophysiological data, realistic neuronal densities, noradrenergic and long-range inputs, and certain connectivity details, including depth dependence and subcellular distribution of synapses. Cortical circuit models with the required level of detail exist for other cortical regions, such as the somatosensory cortex,^36-38^ the visual cortex,^39^ and the auditory cortex.^40^

We have now developed a multiscale mouse M1 model that incorporates recent experimental data and predicts *in vivo* layer- and cell-type-specific behavior-dependent responses. The model simulates a cylindric cortical volume with over 10,000 neurons and 30 million synapses. We focused on the role of L5 excitatory neurons, using detailed models of L5 IT and PT neurons with full dendritic morphologies of 700+ compartments based on anatomical cell reconstruction and ion channel distributions optimized to *in vitro* experimental measures. The task of integrating experimental data into the model required us to develop several methodological techniques for brain circuit modeling, including (1) specifying connections as a function of normalized cortical depth (NCD)—from pia to white matter—instead of by layer designations, with 100–150 *μ*m resolution; (2) identifying and including specific dendritic distributions associated with particular inputs using features extracted from subcellular channelrhodopsin-2-assisted circuit mapping (sCRACM) studies^15,30,41^; and (3) utilizing a high-level declarative modeling tool, NetPyNE, to develop, simulate, optimize, analyze, and visualize the circuit model.^42^ To better understand M1 population dynamics, we employed dimensionality reduction methods to explore behavior-related latent dynamics, calculated the exact contribution of different populations to local field potentials, and developed an approach to estimate layer- and cell-type-specific synaptic drive to different neural populations.

Our M1 model exhibited neuronal firing rates and oscillations that depended on cell class, layer and sublaminar location, and behavioral state, consistent with *in vivo* M1 data. We modeled behavioral changes (quiet wakefulness vs. movement) by modifying noradrenergic inputs from LC and motor thalamus inputs. Our cortical model also captured the effects of experimental manipulations, including blocking of noradrenergic receptors and motor thalamus inactivation. The model provided different multiscale mechanistic hypotheses for the observed behavioral deficits, linking noradrenaline blockade to cell-type-specific changes in *I*_h_ and potassium conductances and the subsequent changes in neuronal firing patterns. The simulations generated experimentally testable quantitative predictions about layer- and cell-type-specific responses for the different behavioral states and experimental manipulations. Two key model predictions were that stronger thalamic and noradrenergic inputs are required to activate the deeper (associated with motor execution) vs. superficial L5B PT neurons and that L5 interneurons support switching between PT and IT output through mutual disynaptic inhibition. Simulations also shed light on M1 circuitry and biophysical mechanisms associated with dynamic aspects of behavior-related activity, including PT cells predominantly mediating an increase in gamma physiological oscillations recorded in L5 local field potentials during movement.

Our model is freely available as a community resource for research and can be updated and extended with new data, including those from the recently released M1 multimodal cell census and atlas by the BRAIN Initiative Cell Census Network.^24^

## RESULTS

### Overview of model development and simulations

**Figure 1.**
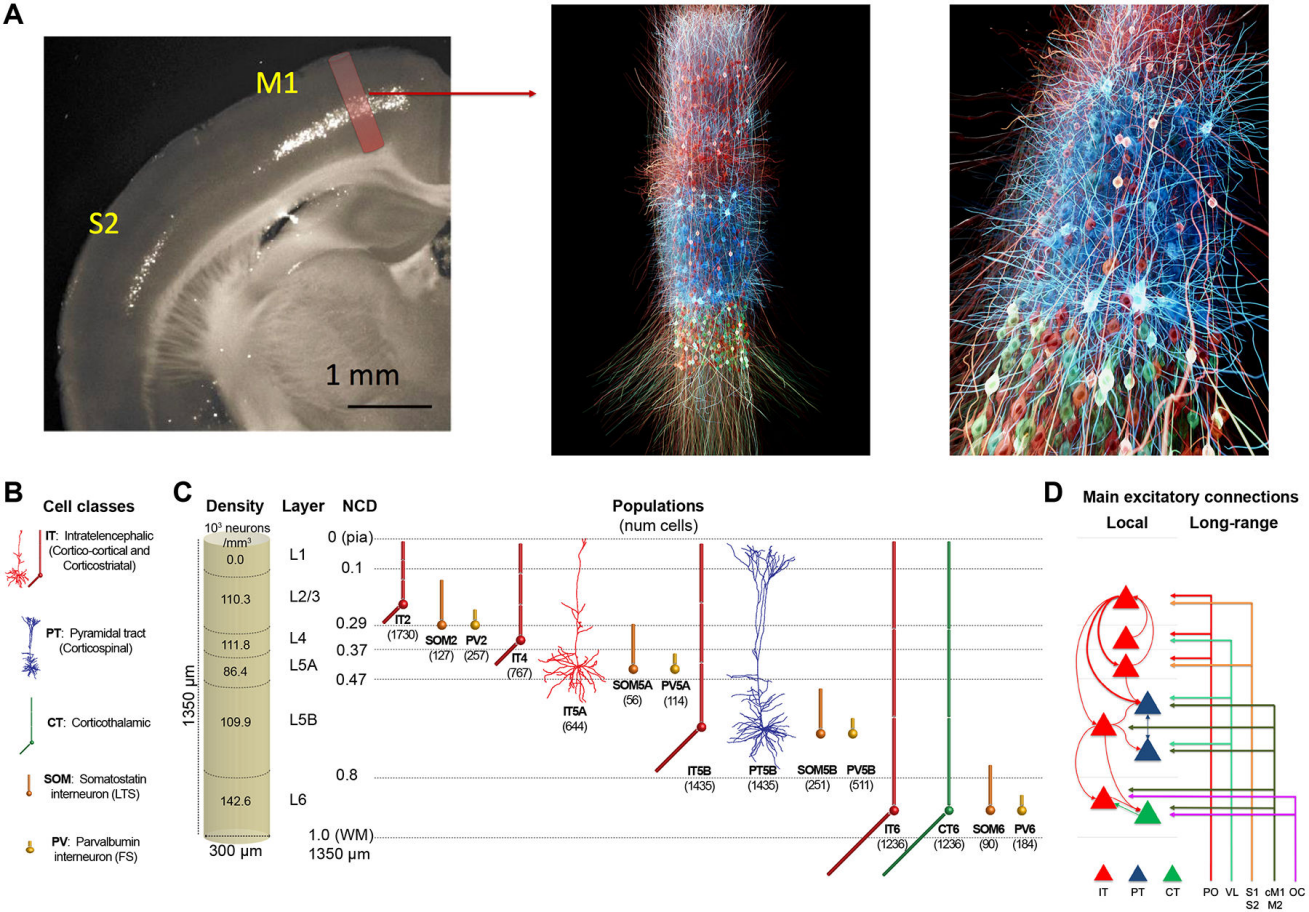
M1 microcircuit model: 3D visualization, connectivity, dimensions, and neuronal densities, classes, and morphologies (A) Left panel: epifluorescence image of coronal brain slice of a mouse showing M1 and S1 regions, with approximate anatomical location and volume of simulated cylindrical tissue (adapted from Suter et al.^47^). Middle and right panels: 3D visualization of M1 network, showing the location and stylized morphologies of 20% of excitatory IT (red), PT (blue), and CT (green) cells, and snapshot of simulated activity with spiking neurons in brighter color (visualization by http://nicolasantille.com). (B) Cell classes. IT5A and PT5B neurons are simulated in full morphological reconstructions. Other excitatory types and inhibitory neurons use simplified models with 2–6 compartments. All models are conductance based with multiple ion channels tuned to reproduce the cell’s electrophysiological characteristics. (C) Dimensions of simulated M1 cylindrical volume with overall cell density per layer (left) and cell types and populations (right). Number of cells in each population shown in brackets; left shows L1–L6 boundaries with normalized cortical depth (NCD) from 0 = pia to 1 = white matter. (D) Schematic of main local and long-range excitatory connections (thin line: medium; thick line: strong). Note the unidirectional projections from IT to PT cells, with a particularly strong projection arising from L2/3. IT, intratelencephalic cells; PT, pyramidal-tract cells; CT, corticothalamic cells; PO, posterior nucleus of thalamus; VL, ventrolateral thalamus; S1, primary somatosensory cortex; S2, secondary somatosensory cortex; cM1, contralateral M1; M2, secondary motor cortex; OC, orbital cortex; PV, parvalbumin interneurons; SOM, somatostatin interneurons.

We implemented a biophysically realistic model of the mouse M1 microcircuit representing a cylindrical volume of 300 *μ*m diameter (Figure 1). The model included over 10,000 neurons with 35 million synapses. We derived cell properties, locations, and local and long-range connectivity from a coherent set of experimental data. The available experimental data were particularly detailed for two L5 populations that were the focus of this study: PT corticospinal cells and IT corticostriatal cells. One innovative feature in the network presented here was to include a L4 for motor cortex, consistent with its recent characterization.^24,43-45^ We developed the model using the NetPyNE^42^ modeling tool and the NEURON simulation engine.^46^ Over 20,000 simulations and 8 million high-performance computing (HPC) cluster core hours were required to build the model and obtain the results shown. One second of simulation time required approximately 96 core h of HPC time. We developed the model by progressively adding features and fixing issues, evaluating each version via simulations. As a final step, we employed an iterative grid search method to optimize under-constrained model parameters to identify simulations that produced physiologically reasonable firing patterns across populations. Details of our model building and optimization approach can be found in the STAR Methods.

As expected from results in other systems, there was no single “correct” model that produced these realistic firing patterns but rather a family of models (degenerate parameterization) that were within the parameter ranges extracted from experimental data.^48-51^ From these, we selected one base model, representing a single parameter set, to illustrate in this article. This base model was tested for robustness by changing randomization settings to generate a model set, analyzing raw and average data from 25 simulations: 5 random synaptic input seeds × 5 random connectivity seeds. This can be considered analogous to testing multiple trials and subjects in an experimental setup. The full model set showed qualitatively similar results with low variance in bulk measures (population rates, oscillation frequencies) for changes in randomization settings.

We used the base model and model set to characterize firing and local field potential (LFP) patterns in response to different levels of long-range inputs and noradrenaline (NA) neuromodulation associated with different behavioral states and experimental manipulations of mouse M1 *in vivo*^26^ (see Table S1). Long-range inputs originated from seven regions: posterior nucleus of thalamus (PO), ventrolateral thalamus (VL), primary somatosensory cortex (S1), secondary somatosensory cortex (S2), contralateral M1 (cM1), secondary motor cortex (M2), and orbital cortex (OC). In the context of this model, the VL will be equivalent to the motor thalamus (MTh) for consistency with the experimental study.^26^ The two behavioral states corresponded to quiet wakefulness and self-paced, voluntary movement. Each of these states was simulated under three different experimental manipulations mimicking those previously performed *in vivo*^26^: control, MTh inactivation, and blocking NA input from the LC using NA receptor antagonists (NA-R block). The effect of changes in NA neuromodulation were initially simulated by altering *I*_h_ conductance in PT cells (see Table S1 and STAR Methods), consistent with *in vitro* findings.^23^ Results are presented both in terms of cell class and cell population. We focused on three excitatory classes, IT, PT, and CT, and two inhibitory classes, parvalbumin-expressing fast-spiking basket cells (PV) and somatostatin-expressing low-threshold spiking cells (SOM). Cell populations are defined by both class and layer (e.g., IT5A indicates IT class in L5A; CT6 is CT class in L6). We use our results to explain and predict the response of M1 circuitry under the different behavioral states and experimental manipulations.

### M1 firing dynamics during quiet wakefulness (spontaneous activity)

**Figure 2.**
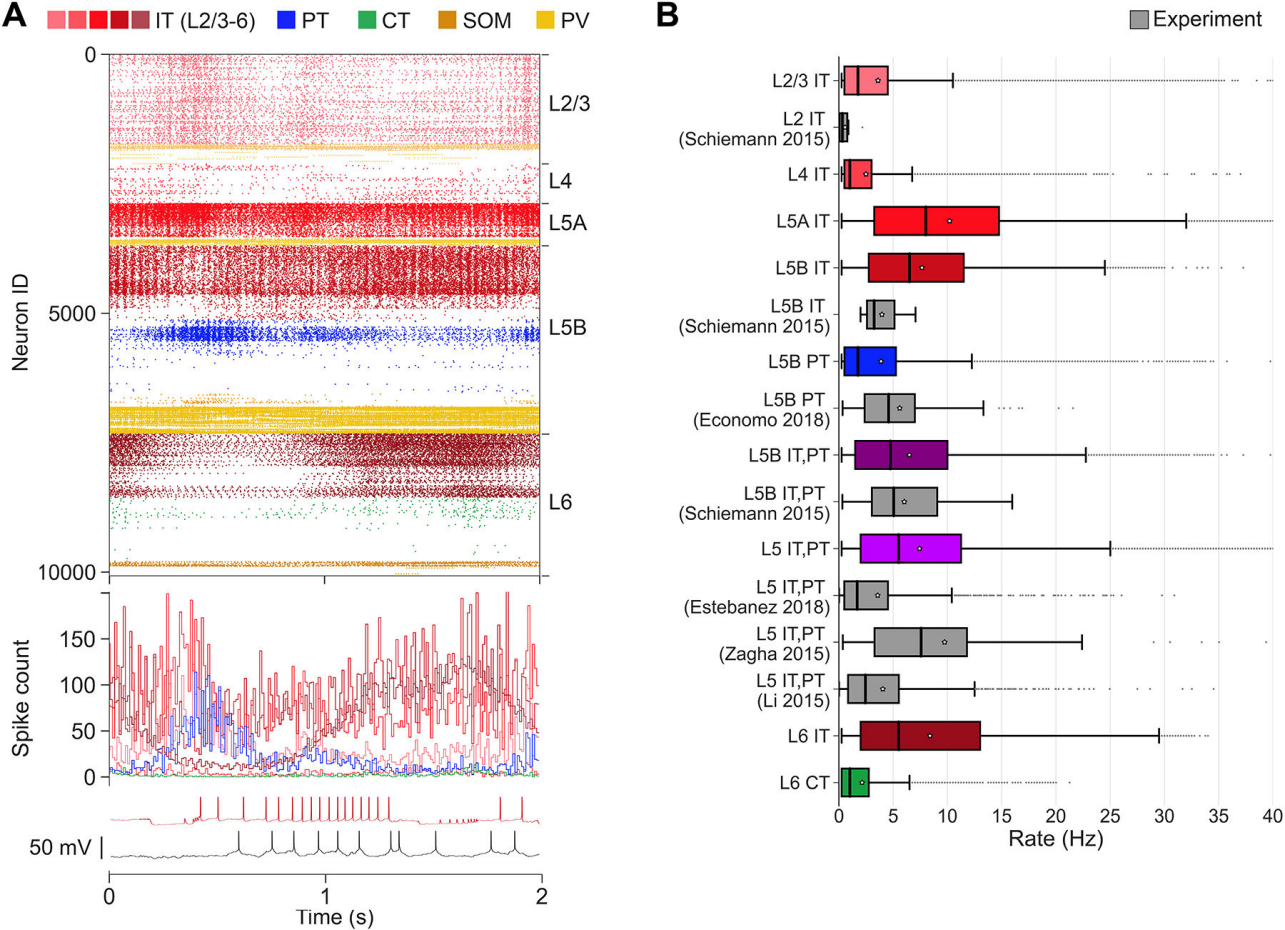
M1 cell-type- and layer-specific firing dynamics during quiet wakefulness and control condition (spontaneous activity) The quiet state was simulated by driving the network with background activity (≤5 Hz) from all long-range inputs, and medium level *I*_h_ (75%) in PT cells (low NA modulation). (A) Top: raster plot of mid-simulation activity (2 s of base model simulation shown; cells grouped by population and ordered by cortical depth within each population). Middle: spike count histogram for excitatory populations (5-ms time bins). Bottom: example model (red) and experiment (black) IT5B somatic membrane voltage (action potentials have been truncated to highlight subthreshold voltage). (B) Firing rates statistics (boxplots) for different cell types and layers in the model set (color bars) and experiment (gray bars). Boxplots show the minimum, lower quartile, median and mean (white star), upper quartile, and maximum values.

We characterized *in vivo* spontaneous activity in the base model. This was simulated based on the expected background drive of ≤5 Hz from all long-range inputs and low NA input resulting in medium-level *I*_h_ (75%) in PT cells (Figure 2).^52,53^ These properties were consistent with the low NA and MTh input associated with the quiet wakefulness state in our mouse M1 *in vivo* study.^26^ We validated the M1 model cell-type- and layer-specific firing rates against available *in vivo* experimental data from mouse motor cortex^26,54-57^ (Figure 2B). We note that these experimental data were not used during parameter optimization. All population mean and median firing rates ranged between 0.1 and 10 Hz, and maximum rates (excluding outliers) were below 35 Hz, for both model and experiment. More specifically, we compared L2/3 IT (median ± interquartile range [IQR]: model = 1.8 ± 4 Hz; experiment [exp] = 0.3 ± 0.7 Hz), L5B IT (model = 6.5 ± 8.8 Hz; exp = 3.2 ± 2.5 Hz), and L5B PT (model = 1.8 ± 4.8 Hz; exp = 4.6 ± 4.6 Hz). Since certain studies did not distinguish between cell types or sublayers, we also compared L5B IT+PT (model = 4.8 ± 8.5 Hz; exp = 5.1 ± 6 Hz) and L5 IT+PT (model = 5.5 ± 9.2 Hz; exp1 = 1.7 ± 4 Hz; exp2 = 7.6 ± 8.5 Hz; exp3 = 2.4 ± 4.7 Hz). Significant statistical differences among population firing rates from different studies are expected. The model results were in range of the experimental values. An example is L5 IT+PT, where two experimental datasets were statistically significantly different (exp1 = 1.7 ± 4 Hz; *n*_*exp*1_ = 1,642; exp2 = 7.6 ± 8.5 Hz; *n*_*exp*1_ = 64; p = 1.2e – 14; rank-sum test with Bonferroni correction), while this was not the case when comparing the L5 IT+PT model with the experiment (model = 5.5 ± 9.2 Hz; *n*_*model*_ = 61,305; exp2 = 7.6 ± 8.5 Hz; *n*_*exp*2_ = 64; p = 0.86; rank-sum test with Bonferroni correction). Overall, these results indicate that the range of firing rates and variability in the model was consistent with that of *in vivo* mouse data.

Activity patterns depended not only on cell class and cortical layer but also on sublaminar location. This supports the importance of identifying connectivity and analyzing activity by NCD in addition to layer. For example, L5B PT firing rates decreased with cortical depth (Figure 2A), consistent with depth-weighted targeting from L2/3 IT projections.^6,17^ This firing pattern was consistent across network variations with different wiring and input randomization seeds. L5A/B IT exhibited similar cortical-depth-dependent activity. L2/3 and L4 IT populations showed overall lower rates than L5 IT, consistent with weaker excitatory projections onto these populations from local M1^17,43^ and from long-range inputs.^30,43,58^ Although the model anatomical connectivity was empirically constrained, population responses are not fully defined by the anatomy but emerge from the complex dynamical interplay across different excitatory and inhibitory populations.

### M1 firing dynamics during movement

**Figure 3.**
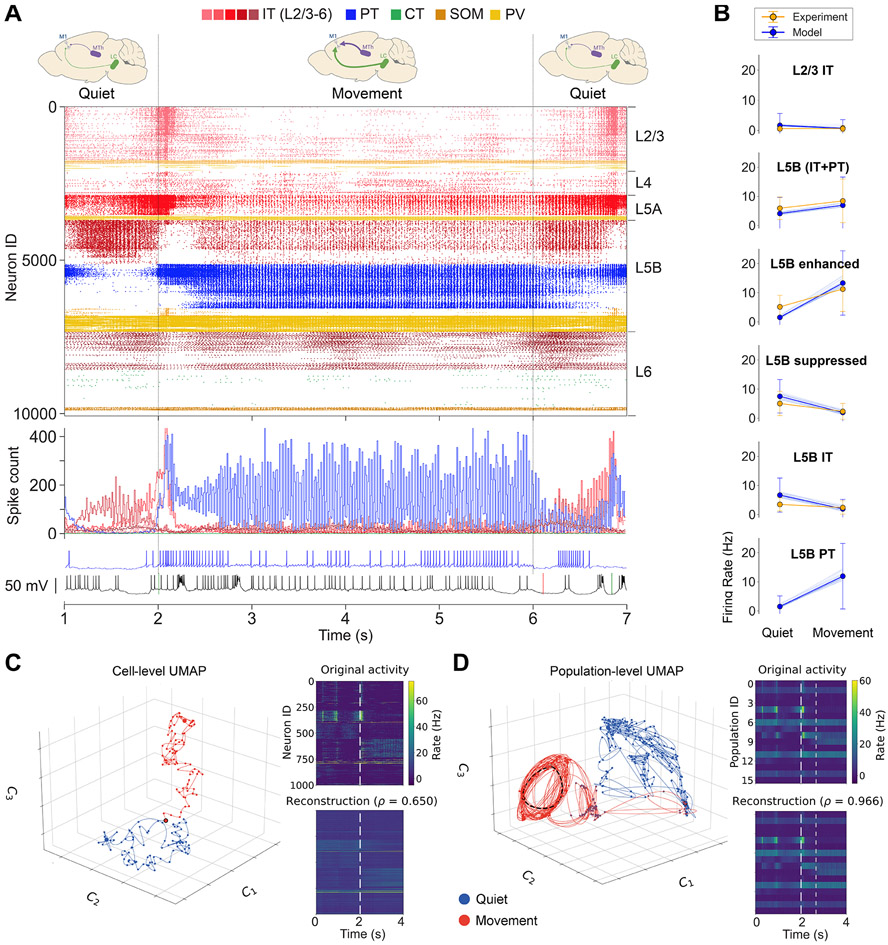
M1 cell type- and layer-specific firing dynamics during quiet wakefulness and movement The movement state was simulated by driving the network with increased activity (0–10 Hz) from motor thalamus, background activity (%5 Hz) from the 6 remaining long-range inputs, and reducing *I*_h_ to 25% in PT cells (mimicking high NA modulation). (A) Top: raster plot of activity transitioning from quiet (1 s) to movement (4 s) to quiet (1 s) states (6 s of base model simulation shown; cells grouped by population and ordered by cortical depth within each population). Middle: spike count histogram for excitatory populations (5-ms time bins). Bottom: example model PT5B (blue) and experiment (black) somatic membrane voltage (action potentials have been truncated to highlight subthreshold voltage). (B) Firing rate (mean, SD) in different cell populations for model and experiment. Model set includes cell rates of all 25 simulations; the mean rates of each individual simulation are shown as thin blue lines. Statistics were computed across 4 s for each state. (C) Left: low-dimensional representation (UMAP) of the firing rates of 1,000 randomly selected neurons during quiet (0–2 s; blue) and movement(2–4 s; red) states; each dot represents activity within a 25-ms time bin; start and end points indicated with larger dots; behavior transition point indicated with black border. Right: original firing rates of the 1,000 neurons and reconstruction from the 3D UMAP representation. Dotted line indicates behavior transition. (D) Low-dimensional representation (UMAP) of the mean firing rates of the 16 populations during quiet (0–2 s; blue) and movement (2–4 s; red) states; each dot represents activity within a 10-ms time bin; behavior transition period (2,000–2,660 ms) indicated with red circles with blue border; periodic oscillatory pattern indicated with dashed black lines between 4 consecutive points (first and last points are close together), corresponding to an approximately 30-ms period (gamma band). Right: original average firing rates of the 16 populations and reconstruction from the 3D UMAP representation. Dotted lines indicate behavior transition period.

The model reproduced experimental cell-type-specific dynamics associated with movement. The movement state was simulated by increasing long-range inputs from the VL to 0–10 Hz (uniform distribution), and reducing *I*_h_ conductance to 25% in PT cells, to simulate high NA neuromodulatory input from LC. The remaining 6 long-range inputs (PO, S1, S2, cM1, M2, OC) continued to provide background drive (≤ 5 Hz). This resulted in a large increase in L5B PT activity and the development of a strong gamma oscillation (observable in the spike histogram of Figure 3A). PT5B_lower_ neurons, which were largely silent during the quiet state, now exhibited activity similar to that of PT5B_upper_. This is consistent with the involvement of PT, and particularly PT5B_lower_,^57^ in motor control. During movement, the activity of L2/3 IT and L5 IT decreased moderately, whereas L4 IT, L6 IT, and L6 CT firing rates remained similar. There was a transition period from quiet to movement that lasted approximately 600 ms, during which there was a peak in the activity of L5 IT and PT5B_upper_, consistent with efferent motor thalamic projections. This transitory activity peak could also be seen in most of the remaining model set simulations. Although IT2/3 exhibited a similar transition peak in the base model, this was only apparent in seven of the model set simulations, suggesting that this response may depend on other factors such as the phase of the ongoing L2/3 IT delta oscillations.

The model firing rates were generally consistent with the M1 *in vivo* experimental data^26^ across populations and behavioral states (Figure 3B). Both model and experiment L2/3 IT cells exhibited low firing rates during both quiet (mean ± SD: model: ± 3.9 Hz; exp: 0.6 ± 0.7 Hz) and movement states (model: 0.7 ± 2.8 Hz; exp: 0.6 ± 1.1 Hz). The L5B rates, including both IT and PT, were similar in model and experiment and exhibited a similar increase from quiet (model: 4.1 ± 5.5 Hz; exp: 5.9 ± 3.9 Hz) to movement (model: 6.9 ± 9.7 Hz; exp: 8.4 ± 7.5 Hz). Following the experimental data analysis and classification of populations,^26^ we compared rates of cells that exhibited enhanced or suppressed activity from quiet to movement. Both L5B_enhanced_ and L5B_suppressed_ rates exhibited comparable trends in model and experiment. The quiet state L5_Benhanced_ mean ± SD rates were lower in the model than the experiment (model: 1 .5 ± 3.6 Hz; exp: 5.1 ± 4 Hz) but increased to a similar rate during movement (model: 13.2 ± 11.1 Hz; exp: 11.3 ± 7.7 Hz). L5B_suppressed_ model and experiment rates exhibited a similar decrease from quiet (model: 7.5 ± 5.7 Hz; exp: 5 ± 4.2 Hz) to movement states (model: 2 ± 3.1; exp: 2.3 ± 2.7 Hz). L5B IT quiet mean ± SD rates were higher for model vs. experiment (model:6:7± 5.9 Hz; exp: 3.5 ± 2.3 Hz) but also decreased to a similar level during movement (model: 1.9 ± 3.3 Hz; exp: 2.4 ± 2.3 Hz). Model L5B PT rates increased sharply from quiet (1.5 ± 3.6 Hz) to movement (11.9 ± 11.3 Hz). We did not include experiment PT rates in Figure 3B given their small sample size (N = 3) and high variability. However, we note that two of the experiment PT cells showed a decrease from quiet to movement (16–5.6 and 4.7–0.6 Hz), and one showed a similar sharp increase to that of the model (3.5–13.2 Hz). The robustness of the model was evidenced by the small variability across the mean firing rates of the 25 simulations in the model set, each with different randomization seeds (see thin blue lines in Figure 3B).

To study the M1 circuit’s dynamics under different behaviors and experimental manipulations, we used the uniform manifold approximation and projection (UMAP) method to create low-dimensional representations of neuronal activity. We performed dimensionality reduction at two scales: the individual cell level (Figure 3C) and the population level (Figure 3D). For the cell-level approach, we randomly selected 1,000 neurons and calculated their average firing rate every 25 ms for 4 s (2 s for each behavior). The choice of 1,000 was an estimate of the number of neurons in a mouse cortical column that we can simultaneously record using a Neuropixels 2.0 probe.^59^ We used UMAP to project the firing rates of these neurons into a low-dimensional neural manifold for each behavioral state. Although we show data from a single simulation, we combined data from five simulations with different input randomization seeds to perform nonlinear embedding, mimicking different controlled trials in behavioral experiments (see Figure S2).

To help interpret the low-dimensional representation, we employed the Silhouette coefficient, a measure of how dense and well separated one cluster is from another, with values ranging from −1 (incorrectly clustered) to +1 (highly dense and well separated). Values around 0 indicate overlapping clusters.

The low-dimensional representation at the cell level revealed a separation between quiet (blue) and movement (red) behavioral states (Silhouette coefficient = 0.55; Figure 3C). This separation was higher after the initial transition period between behaviors (marked by red circle with black border). The reconstruction of the original high-dimensional activity from the low-dimensional projection achieved a Pearson correlation coefficient of 0.65, which is remarkable considering the drastic increase from 3 to 1,000 dimensions. Despite capturing the overall network dynamics, the reconstruction exhibited a lower temporal resolution, thus missing some time-domain features such as fast oscillations.

For the population-level analysis, we used the UMAP method to perform dimensionality reduction on the average firing rate of 16 neuronal populations (PT5B was split into PT5B_upper_ and PT5B_lower_) at 10 ms time steps (Figure 3D). The low-dimensional representation clearly differentiated between the two behavioral states (Silhouette coefficient = 0.41), except during a transition period between approximately 2,000–2,660 ms (red circles with blue border). After this period, a periodic pattern emerged in the low-dimensional population dynamics, with an approximate period of 30 ms corresponding to a gamma oscillation (Figure 3D, dashed black lines outline the circular trajectory of the oscillation). This is consistent with the L5 oscillatory firing and LFP activity observed during movement. The reconstructed firing activity was almost perfect (Pearson correlation coefficient = 0.97), suggesting that a 3D neural manifold can capture the population-level latent dynamics with minimal information loss.^60^

### M1 L5 LFP oscillations depend on behavioral state

**Figure 4.**
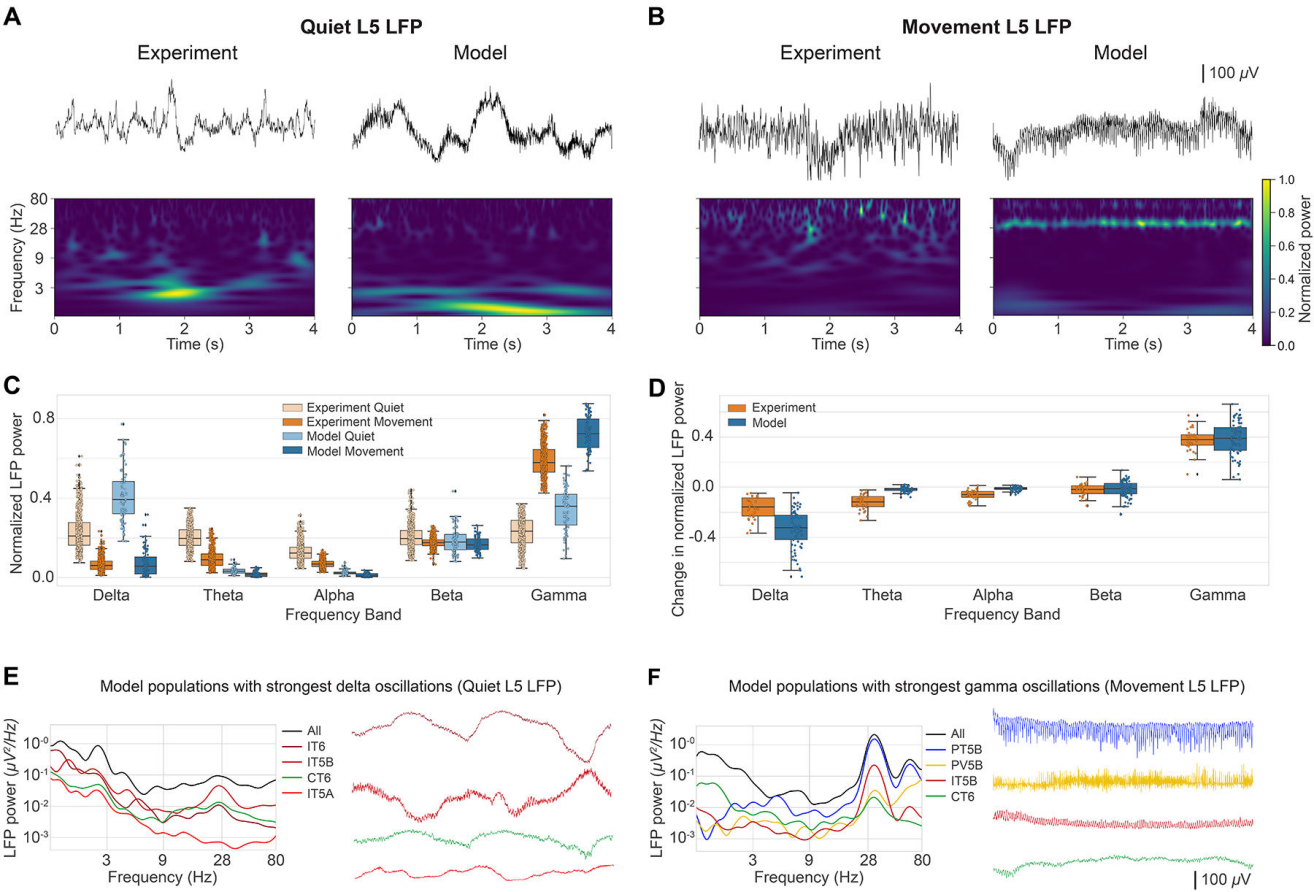
M1 layer 5 LFP oscillations during quiet wakefulness and movement (A and B) Example experiment and model raw LFP signals (top) and spectrograms (middle) during quiet (A) and movement (B) states. (C) Comparison of experiment and model normalized power spectral density (PSD) power across 5 frequency bands during quiet and movement states. (D) Comparison of experiment and model changes in normalized PSD power across 5 frequency bands during quiet and movement states. (E) LFP PSD (left) and waveform (right) of the four populations contributing most to the overall L5 LFP delta band (0–4 Hz) power during the quiet state. (F) LFP PSD (left) and waveform (right) of the four populations contributing most to the overall L5 LFP gamma band (30–80 Hz) power during the movement state. Scale bar representing 100 mV in (B) and (F) also applies to (A) and (E).

*In vivo* studies in mouse vibrissal M1 have shown a decrease in L5 LFP slow oscillations (3–5 Hz) and an increase in gamma oscillations (30–50 Hz) during active whisking.^61^ Here, we reanalyzed the LFP data from Schiemann et al.^26^ to investigate whether similar changes were observed in mouse M1 L5 LFP during the self-paced, voluntary movement task and if these changes were captured by our simulated M1 LFP (Figure 4). Importantly, the model was not tuned to reproduce the experiment LFP during quiet or movement states. Despite this, LFP amplitudes were overall similar in the model and experiment (order of 500 *μ*V). In both experiment and model, the L5 LFP showed weaker slow oscillations (delta) and stronger fast oscillations (gamma) during movement compared with the quiet behavioral state, consistent with previous reports.^61^ This is illustrated in the raw LFP signal and spectrogram examples for experiment and model (Figures 4A for quiet and 4B for movement). Model L5 LFP examples correspond to signals recorded from simulated extracellular electrodes at 800 μm (upper L5B), and for the statistical analysis, we averaged the LFP signals across the 3 depths within L5: 600 (L5A), 800 (upper L5B), and 1,000 μm (lower L5B). The experimental LFP dataset was recorded *in vivo* from L5 extracellular electrodes and preprocessed to remove outliers and potential artifacts (see STAR Methods).

The model reproduced behavioral-dependent differences across different frequency bands of M1 LFP oscillations. To quantify these differences, we calculated the LFP normalized power spectral density (PSD) across the major frequency bands for the experimental and modeling datasets (Figure 4C). To enable comparison, we segmented the experimental data into 4-s samples, matching the duration of the samples from the model dataset, and normalized power density within each sample. Both experiment and model datasets exhibited stronger LFP power at the lower end of the spectrum (delta, theta, and alpha bands) during the quiet state and stronger high-frequency (gamma) LFP power during movement. More specifically, delta (0–4 Hz) power in the quiet state was high in both model vs. experiment (median ± IQR: model: 0.39 ± 0.16; exp: 0.21 ± 0.11; normalized power is dimensionless) but decreased to a similar level during movement (median, IQR: model: 0.06 ± 0.09; exp: 0.06 ± 0.04). Gamma power (30–80 Hz) was stronger during movement for both experiment and model (median, IQR: model: quiet=0.36 ± 0.15 and move=0.72 ± 0.14; exp: quiet=0.23 ± 0.11 and move=0.58 ± 0.11).

The model also reproduced the main changes in LFP power from quiet to movement states (Figure 4D) when comparing paired samples occurring within the same recording (see STAR Methods). Both model and experiment showed results consistent with the previous analysis: from quiet to movement, there was (1) a strong decrease of delta frequency power during movement (model: −0.32 ± 0.19; exp: −0.16 ± 0.14); (2) small changes in theta, alpha, and beta power; and (3) a large increase in gamma power (model: 0.39 ± 0.18; exp: 0.38 ± 0.08). These results further validate that the model captures behavior-related oscillatory dynamics observed in mouse M1 *in vivo*.

To investigate the source of different L5 LFP oscillations, we analyzed the exact contribution of each population (Figures 4E and 4F). For the quiet state, we focused on the predominant delta oscillation and identified IT6, IT5B, CT6, and IT5A as the top contributors to this frequency band (29%, 23%, 8%, and 5% of power in the 0–4 Hz range; Figure 4E). The delta waves generated by these populations were irregular; for example, IT6 peaks were longer than the troughs. Notably, the population LFP signals were not in phase with each other; in fact, the L6 (IT6 and CT6) vs. L5 (IT5B and IT5A) delta oscillations were approximately in antiphase. The irregularity of the overall quiet L5 LFP (Figure 4A) may be explained by the linear combination of the out-of-phase and irregular LFP signals from these four populations.

The LFP gamma oscillation during the movement state (Figure 4B) was primarily caused by the PT5B population, which accounted for 69% of the overall L5 LFP gamma band (30–80 Hz) power (Figure 4F). This is consistent with the increased firing rate and oscillatory activity of PT5B during movement observed in Figure 3. PV5B, IT5B, and CT6 also contributed to the gamma oscillations but only 9%, 8%, and 1% of the power.

### M1 dynamics during MTh inactivation

**Figure 5.**
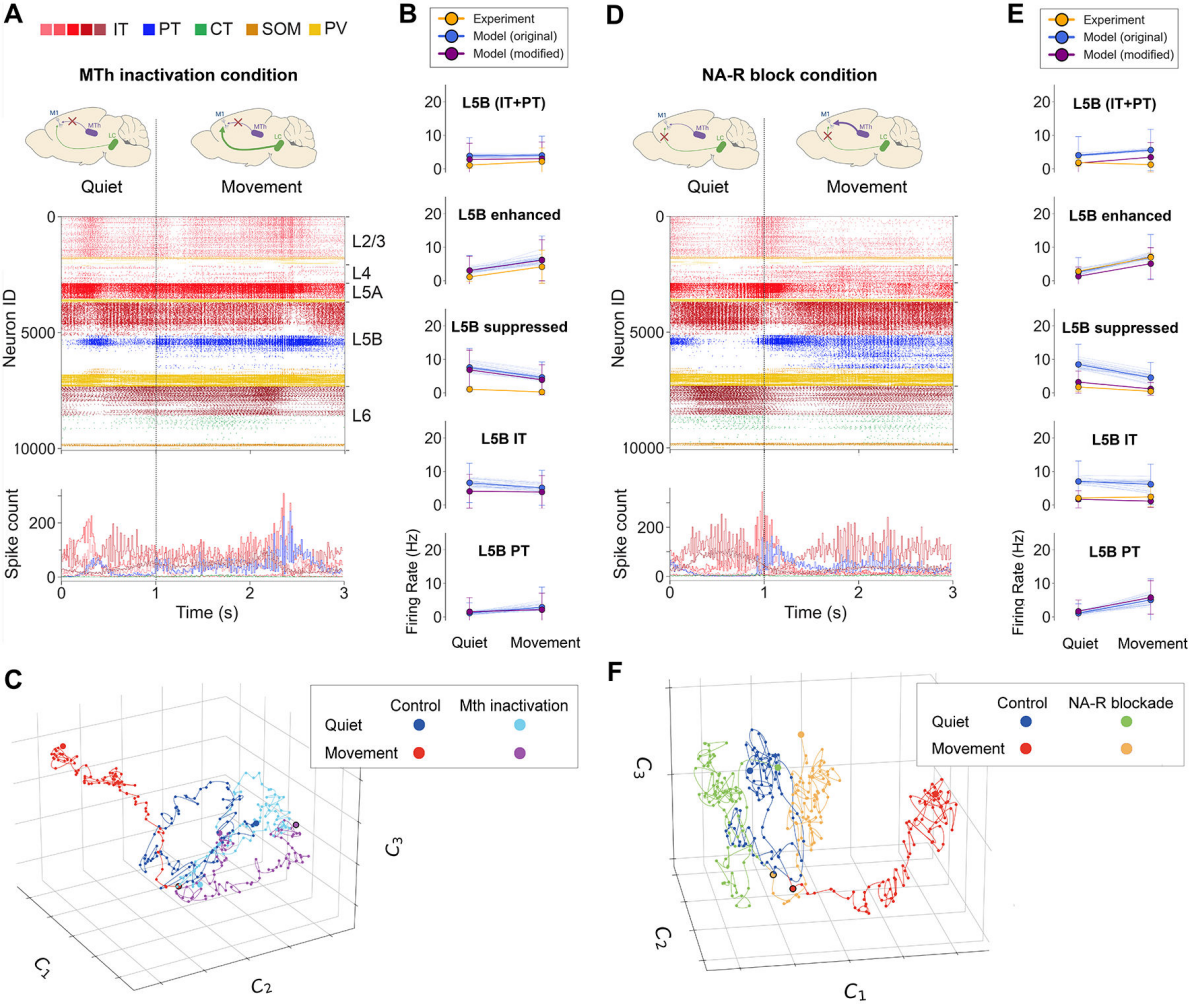
M1 cell-type- and layer-specific firing dynamics during quiet and movement states for MTh inactivation and NA-R-block conditions (A and D) Top: raster plot of activity transitioning from quiet (1 s) to movement (2 s) (3 s of base model simulation shown; cells grouped by population and ordered by cortical depth within each population). Bottom: example model PT5B (blue) and experiment (black) voltage traces. (B and E) Firing rate (mean, SD) in different cell populations for the original model set (blue), modified model (purple), and experiment (orange). The modified model decreased long-range inputs from cM1 and M2 for the MTh inactivation condition and increased K^+^ conductance for the NA-R-block condition. The original model set includes cell rates of all 25 simulations; the mean rates of each individual simulation are shown as thin blue lines. Statistics were computed across 4 s for each state. (C and F) Low-dimensional representation (UMAP) of the firing rates of 1,000 randomly selected neurons during the control condition quiet (0–2 s; blue) and movement (2–4 s; red) states and the MTh inactivation/NA-R-block condition quiet (light blue/green) and movement (purple/orange) states. Each dot represents activity within a 25-ms time bin; start and end points indicated with larger dots; behavior transition point indicated with black border.

To gain insight into the known role of thalamic inputs in regulating M1 output,^27,28^ we simulated an experimental manipulation described in our *in vivo* study^26^ consisting of blocking motor thalamic input to M1 by local infusion of the *GABA*_*A*_ receptor agonist muscimol into the VA/VL complex. Our computational model captured several features of inactivating MTh inputs to M1. MTh inactivation was simulated by removing VL input. The other six long-range background inputs (PO, cM1, M2, S1, S2, OC) remained. Under this condition, the change from quiet to movement states only involved reducing *I*_h_ conductance from 75% to 25% in PT cells, simulating high NA neuromodulatory input from the LC. The decrease in movement-associated L5B activity (mean ± SD: control: 6.9 ± 9.7 Hz; MTh inactivated [inact]: 4 ± 5.7 Hz) after MTh inactivation (Figures 5A and 5B) was consistent with that observed experimentally (control: 8.4 ± 7.5 Hz; MTh inact: 2.2 ± 4 Hz).^26^ The model also captured the strong reduction in movement-associated responses of L5B_enhanced_ cells following MTh inactivation (model control: 13.3 ± 11.1 Hz; MTh inact: 6.3 ± 7.1 Hz; exp control: 11.3 ± 7.7, MTh inact: 4.2 ± 4.9). The decrease in model L5B rates was caused by a strong reduction in PT rates (control: 11.9 ± 11.3 Hz; MTh inact: 2.9 ± 6 Hz). MTh inactivation resulted in a particularly strong reduction in the movement-associated PT5B_lower_ population, which was practically silenced.

However, the model did not adequately capture some effects of MTh inactivation on M1 L5B, particularly during the quiet state, highlighting potential improvements to the model. Specifically, MTh inactivation led to a reduction of quiet state L5B firing rate (mean ± SD: control: 5.1 ± 3.9 Hz; MTh inact: 1.1 ± 1.1 Hz), as well as L5B_suppressed_ rates, which was not observed in our model. This discrepancy could be due to the lack of interaction between long-range inputs in the model, preventing it from capturing the effects of MTh inactivation on other regions (e.g., M2) that in turn provide input to M1 (see discussion for more details and alternatives). We evaluated this by modifying our original model of MTh inactivation by reducing the activity of other cortical long-range inputs (cM1, M2). The modified model better reproduced experimental L5B results (see Figure 5B, purple lines) both for the quiet state (original [orig] model: 3.9 ± 5.4; modified model for MTh inactivation: 2.8 ± 4.8; exp: 1.1 ± 1.1 Hz) and movement state (original model: 4 ± 5.7 Hz; modified model for MTh inactivation:± 5.0; exp: 2.2 ± 4 Hz).

We explored the effect of MTh inactivation on the circuit latent dynamics using the same cell-level dimensionality reduction approach as before (Figure 5C). After MTh inactivation, both quiet and movement neural trajectories were relatively similar and resembled the quiet state of the control condition (see Table S2 for the Silhouette coefficients between all cluster pairs). This may correlate with behavioral changes associated with MTh inactivation such as deficits in posture and voluntary motor control.^28^ A similar pattern was observed for the MTh inactivation low-dimensional trajectories in the modified model with decreased long-range inputs from both cM1 and M2 (Figure S3).

### M1 dynamics during NA-R blockade

We then explored the role of NA neuromodulation in the model, motivated by our *in vivo* study in which blocking NA inputs through local infusion of NA-R antagonists resulted in reduced motor coordination.^26^ A recent study also showed that inactivating NA axons in M1 affected motor execution.^62^ The model reproduced key aspects of the experimental M1 L5B responses under NA-R blockade (denoted as the NA-R block condition). NA-R block was initially simulated by increasing *I*_h_ to the *in vitro* level (100% *I*_h_ conductance in PT cells), reflecting no NA neuromodulation from LC. Long-range inputs from seven cortical and thalamic regions were unchanged from the control condition. Under NA-R block, the change from quiet to movement states only involved increasing the firing rate of MTh inputs. NA-R block resulted in decreased L5B activation during movement compared with the control condition (Figures 5D and 5E) (mean ± SD: control: 6.9 ± 9.7 Hz; NA-R block: 5.6 ± 6.2 Hz), particularly in the PT5B population (control: 11.9 ± 11.3 Hz; NA-R block: 5.1 ± 6.3 Hz). *In vivo* experiments showed a more pronounced decrease in L5B firing rates during movement (control: 8.4 ± 7.5 Hz; NA-R block: 1.3 ± 2.2 Hz).

These discrepancies between experiment and model suggested that the model could be improved to capture additional effects of NA LC input. One known effect of NA that was lacking in the model was the modulation of potassium (K^+^) conductance.^63,64^ We therefore modified the model during the NA-block condition by increasing K^+^ conductance by 50% in all excitatory cell types, in addition to the existing increase of *I*_h_ in PT cells. The modified model better captured the experimental responses during the NA-block condition (see Figure 5E, purple lines). More specifically, the mean firing rates of L5B, L5 IT, and L5B_suppressed_ were lower for both the quiet (L5B IT: orig model: 7 ± 6.1; modified model for NA-R block: 1.7 ± 2.6; exp: 2.0 ± 0.7 Hz) and movement (L5B IT: orig model: 6.2 ± 6.0; modified model for NA-R block: 1.1 ± 1.9; exp: 2.4 ± 3.0 Hz) responses, more closely matching those recorded *in vivo*.

The low-dimensional representation of the NA-R block condition revealed a similar pattern to the MTh inactivation condition (Figure 5F). Both NA-R-block quiet and movement trajectories resembled the control quiet trajectory (see Table S2 for the Silhouette measures between all clusters) but were clearly distinct from the control movement trajectory. This is consistent with the reduced L5B response and behavioral deficits observed experimentally for the NA-R-block manipulation during movement.^26^ A similar pattern can be observed for the low-dimensional representation of the NA-R-block trajectories in the modified model with increased K^+^ conductance (Figure S3).

### Motor thalamic and NA inputs affect L5B dynamics in a cell-type- and sublayer-specific manner

**Figure 6.**
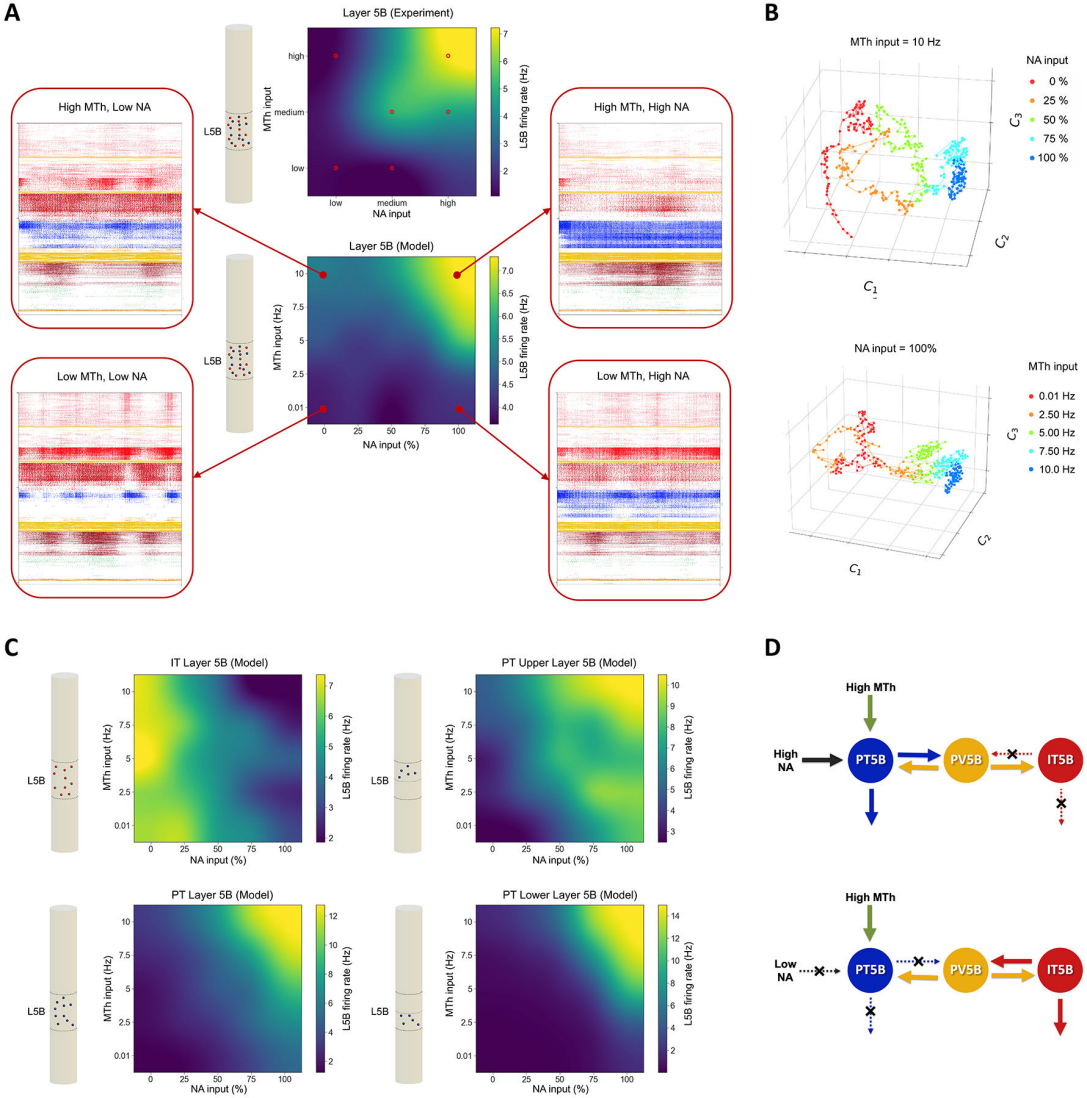
Cell-type- and sublayer-specific effects of MTh and NA input levels on L5B dynamics (A) Mean L5B firing rate response of experiment (top) and model (bottom) to different levels of MTh and NA inputs. Experimental firing rates derived from control, MTh inactivation, and NA-R-block conditions are indicated with small red circle (remaining values were extrapolated). All model firing rates were derived from simulations using the specified MTh and NA inputs. Insets show the model raster plots corresponding to the corner values of MTh and NA input (4 s; cell type colors and layers same as raster plots in Figures 2, 3, and 5). (B) Low-dimensional representation (UMAP) of the firing rates of 1,000 randomly selected neurons across different levels of NA input with fixed high MTh input (top) and across different levels of MTh input with fixed high NA input (bottom). Each trajectory represents 2 s of activity, and each dot represents activity within a 25-ms time bin. (C) Same as in (A) but for different L5B cell types and subpopulations (IT, PT, PT5B_upper_, and PT5B_lower_), each of which showed highly specific response patterns to MTh and NA. Schematic cylinders illustrate the cell type (IT = red; PT = blue) and layer analyzed. (D) Schematic of hypothesized NA input and mutual disynaptic inhibitory pathway mediating the switching between IT- and PT-predominant output modes.

Our model reproduced the pattern of M1 L5B *in vivo* responses observed experimentally for different levels of MTh and NA inputs and provided insights and predictions of how the different L5B subpopulations respond and interact (Figure 6). The experimental and modeling results reported so far suggest that M1 L5B responses depend strongly on MTh and NA inputs. Figure 6A shows the mean L5B firing rates (top) from experiment and model (bottom) as a function of the strength of these inputs, illustrating that MTh and NA inputs moderately increase the L5B response, but both are simultaneously required to trigger high L5B activity. Both experiment and model exhibit a similar response pattern, progressively increasing with MTh and NA, and a similar range of L5B firing rates. To provide a better intuition of the full circuit model dynamics, we also included the spiking raster plots for the four conditions with minimum and maximum MTh/NA values (Figure 6A).

Dimensionality reduction of the network dynamics revealed low-dimensional structures with clustering of data points according to the input level of NA and MTh input level (Figure 6B; Silhouette coefficients included in Tables S3 and S4). These representations depict an approximate monotonic and separable response with increasing levels of NA and MTh input, primarily along a single dimension (C1). This suggests that the low-dimensional neural manifold can capture the distinct latent dynamics of M1 in response to different levels of NA neuromodulation and long-range MTh input. The results also predict a similarity in the M1 latent dynamics response pattern to changes in MTh and NA inputs.

The model also revealed highly specific and distinct activity patterns for the different L5B cell types and sublayers (Figure 6C). When PT5B fired strongly due to high MTh and NA, supragranular IT2/3 and IT5A populations exhibited low activity (see Figure 6A, raster plots), consistent with the predominant involvement of PT cells in motor execution.^18^ Interestingly, L5B IT cells exhibited an inverse response pattern to NA compared with L5B PT and with the overall L5B response (Figure 6C), showing decreased firing with increases in NA inputs, and a largely constant response to MTh inputs. The response to NA is consistent with the low levels of *I*_h_ in L5B IT cells.^23^ We hypothesize that the inverse response to NA between L5B IT and PT cells could be caused by mutual inhibition mediated via L5 interneurons (see schematic in Figure 6D). L5B PT cells showed higher peak firing rates than IT (12.8 vs. 7.4 Hz), thus dictating the overall L5B response pattern (Figure 6A) and overshadowing the L5 IT inverse pattern.

The model also exposed sublaminar differences in L5B PT response, with PT5B_lower_ exhibiting increased minimum and maximum rates when compared with PT5B_upper_ (0 – 15 vs. 3 – 10 Hz). The PT5B_lower_ activation threshold was also higher than that of PT5B_upper_; that is, PT5B_lower_ required higher MTh and NA inputs to respond strongly. This suggests that PT5B_upper_ would activate first followed by a delayed response from PT5B_lower_, as inputs associated with motor execution accumulate and reach a threshold. These results align with the suggested role of PT5B_upper_ in movement preparation and PT5B_lower_ cells in movement initiation.^57^

### Cell-type-specific presynaptic inputs to L5B subpopulations during quiet vs. movement behaviors

**Figure 7.**
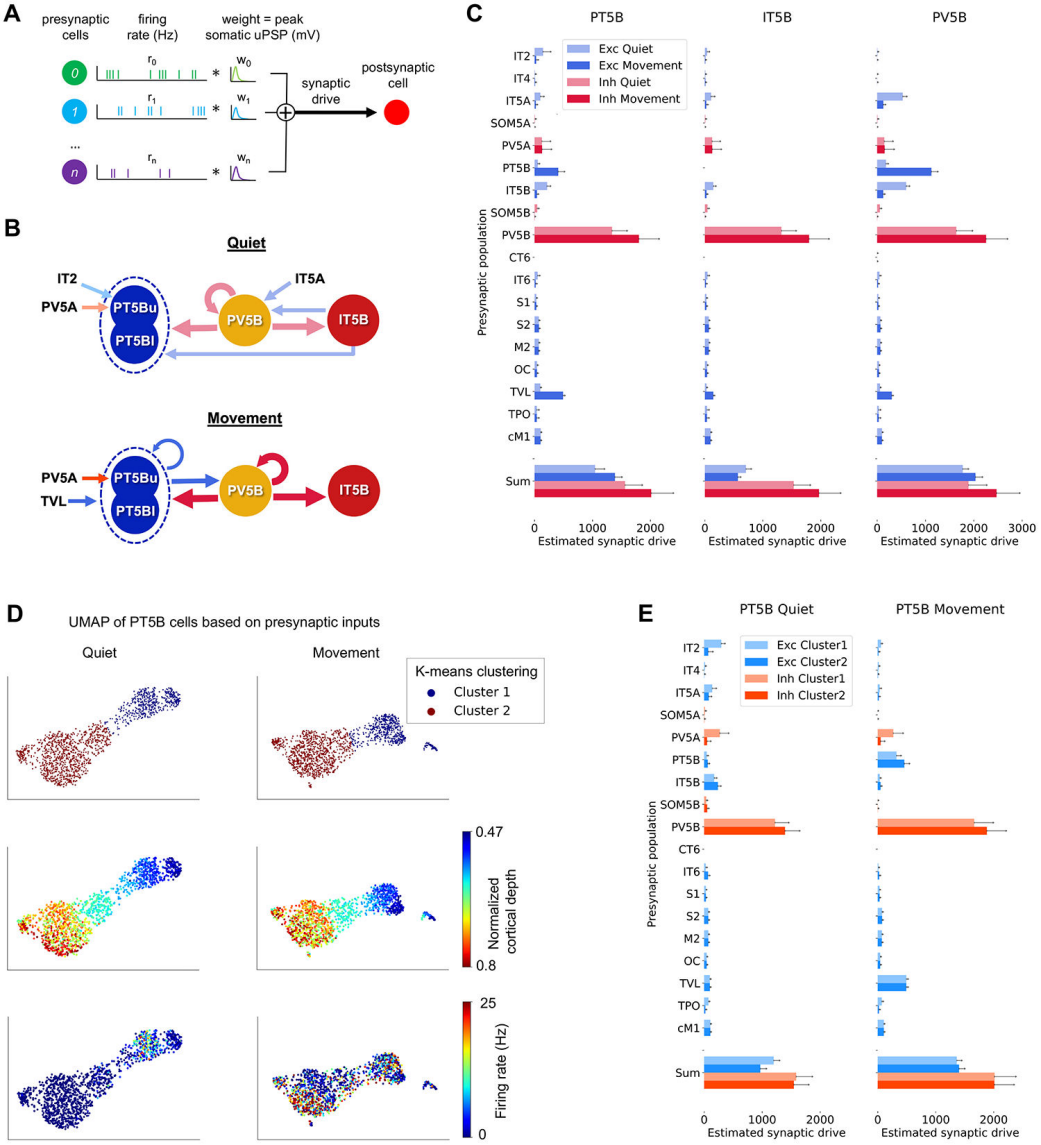
Cell-type-specific presynaptic inputs to L5B subpopulations during quiet vs. movement behaviors (A) Schematic illustrating the calculation of synaptic drive for a postsynaptic neuron: the presynaptic cell firing rate (Hz) times the synaptic weight representing the somatic unitary postsynaptic potential (uPSP) peak amplitude (mV), summed across all presynaptic cells. (B) Schematic summarizing the major synaptic inputs to L5B populations based on results from (C) and (E). Arrow widths represent estimated synaptic drive (not to scale); arrow colors follow legends in (C) and (E); PT5Bu and PT5Bl represent PT5B_upper_ and PT5B_lower_, which largely overlap with clusters 1 and 2. (C) Excitatory and inhibitory estimated synaptic drive from presynaptic populations to L5B populations. Error bars represent SD across the postsynaptic population neurons. (D) UMAP low-dimensional representation of synaptic inputs to PT5B neurons (dots) for quiet and movement states, colored according to K-means clusters (top), normalized cortical depth (middle), and postsynaptic cell firing rate (thresholded at 25 Hz) (bottom). (E) Excitatory and inhibitory estimated synaptic drive from presynaptic populations to the two clusters of PT5B cells during the quiet and movement states. Error bars represent SD across the postsynaptic population neurons.

To better understand why the firing patterns of different populations change during the quiet and movement behaviors, we analyzed the specific synaptic inputs that drive these cells (Figure 7). We focused on L5B PT, IT, and PV cell types as our previous results suggested that they play a key role in behavior-dependent changes. Analyzing inputs to these populations also allowed us to further explore the L5B disynaptic inhibitory pathway hypothesis depicted in Figure 6D. This analysis leverages the model’s ability to access the activity and connections of all neurons to reveal the contributions of different types of input, such as excitatory vs. inhibitory, local vs. long range, and even across specific cell types. Our approach for estimating synaptic drive is illustrated in Figure 7A and detailed in the STAR Methods, where we also explain its limitations.

Figure 7B summarizes the main presynaptic populations driving PT5B, IT5B, and PV5B during quiet and movement states, based on the detailed analysis of estimated synaptic drive (ESD) shown in Figures 7C-7F. The firing rate of most PT5B cells (85%) was enhanced during movement, primarily due to stronger long-range VL and local recurrent PT5B synaptic inputs (ESD change from quiet to move: VL = 5.0×, PT5B = 7.3×). Although some excitatory inputs decreased and some inhibitory inputs increased, total excitation increased more than total inhibition (ESD change from quiet to move: excitatory [exc] = 1.33×, inhibitory [inh] = 1.27×). Most IT5B cells (83%) showed suppressed firing rates during movement, mainly due to increased PV5B and decreased IT5A and IT5B synaptic inputs (ESD change from quiet to move: PV5B = 1.38×; IT5A/B = 0.22×).

Analyzing synaptic inputs to both PT5B and IT5B revealed an important role for PV5B in modulating behavior-dependent dynamics. PV5B provided strong inhibitory inputs to IT5B and PT5B during both behavioral states. However, the primary synaptic driver of PV5B cells notably switched from IT5A and IT5B during the quiet state to PT5B during movement (percentage of PV5B total excitatory ESD during quiet: IT5A/B = 60%, PT5B = 10%; movement: IT5A/B = 13%, PT5B = 55%). This provides further insights into the disynaptic pathway hypothesis outlined in Figure 6D: during the quiet state, IT5B activity predominates, primarily driving PV5B, which in turn suppresses PT5B; during movement, PT5B activity increases due to VL excitatory inputs, making PT5B the main driver of PV5B, which in turn suppresses IT5B activity. Remarkably, the predominant L5B excitatory population (IT5B during quiet or PT5B during movement) also receives strong PV5B inhibitory inputs. This suggests that PV5B not only suppresses the competing population but also interacts in a temporally precise manner with the predominant excitatory population to produce the observed gamma oscillatory pattern. This is consistent with the pyramidal interneuron network gamma (PING) mechanism for cortical gamma oscillations.^65^

To investigate whether the input patterns to PT5B subpopulations were different, we used dimensionality reduction (UMAP) to analyze PT5B cells based on their ESD from presynaptic populations (Figure 7D). We then applied K-means clustering (with k = 2) to group the data. The clustering of data points was remarkably similar for the quiet and movement states (97% overlap). The low-dimensional UMAP representation was correlated with cortical depth and postsynaptic cell firing rate during the quiet behavior. Clusters 1 and 2 approximately correspond to upper and lower L5B PT cells (Figure 7D). During the quiet behavior, cluster 1 cells fired more strongly than cluster 2 cells (mostly silent) and received stronger synaptic drive from IT2, IT5A, and PV5A but weaker drive from IT5B and PV5B (Figure 7E). This is consistent with the stronger projections from supragranular layers to superficial vs. deeper PT5B cells.^6^ During movement, firing rates were similar between the two clusters, but cluster 1 received stronger input from PV5A and weaker input from PT5B and PV5B (Figure 7E). The stronger PV5A inputs to cluster 1, in both quiet and movement states, can be explained by the proximity of these more superficial PT5B cells to L5A and the distance-dependent connectivity profile of PV cells.^66^

## DISCUSSION

In this work, we have developed a computational model of the mouse M1 microcircuit and validated it against *in vivo* data. Despite inherent limitations due to gaps in the data (see details in the section below), we believe that this constitutes the most biophysically detailed model of mouse M1 currently available comprising the molecular, cellular, and circuit scales. The model integrates quantitative experimental data on neuronal physiology, morphology, laminar density, cell type distribution, dendritic distribution of synapses, and local and long-range synaptic connectivity, obtained from 31 studies, 12 of which come from our experimental laboratories.

To validate the model, we focused on reproducing mouse M1 *in vivo* experimental results across different behavioral states and experimental conditions from a single study.^26^ Simulation results were consistent across multiple random wiring seeds and background input seeds, demonstrating the robustness of the model. The model cell-type-specific spontaneous firing rates, associated with the quiet behavior, were consistent with experimental data from several *in vivo* studies^26,54-57^ (Figure 2). We then simulated activity corresponding to self-paced, voluntary locomotion of the mouse by increasing the MTh and noradrenaline (NA) inputs. Movement-related changes in L2/3 and L5B population firing rates were consistent with those reported *in vivo*, including bidirectional (enhanced vs. suppressed) firing rate changes in distinct L5B pyramidal neuron populations (Figure 3). LFP oscillations emerged spontaneously (no oscillatory inputs) at physiological frequencies, including delta, beta, and gamma. LFP power in L5B shifted from lower (delta) to higher frequency bands (gamma) during movement, consistent with *in vivo* LFP data (Figure 4). Simulation analysis identified the specific populations responsible for generating the delta and gamma oscillations.

We also simulated two experimental manipulations—inactivation of MTh inputs and blocking of NA-Rs—that resulted in changes in cell-type-specific activity in L5B that matched those measured experimentally (Figure 5). Some *in vivo* results were better captured by a modified model version in which MTh inactivation affected other long-range cortical inputs, and NA modulation also affected K^+^ conductances. We used the model to systematically explore the interaction between MTh and NA inputs and predict M1 output at the level of individual cell types at sublaminar resolution. Results captured the general pattern and response amplitudes measured *in vivo*, supporting the hypothesis that both high MTh and NA inputs are required for self-paced voluntary movement-related L5B activity (Figure 6).

The model predicted a predominant role for PT cells in dictating L5B responses during movement, with PT5B_lower_ providing the strongest response but only when both MTh and NA inputs were high enough, that is, PT5B_lower_ exhibited the highest response threshold. L5B IT cells exhibited an opposite but lower amplitude pattern due to PT-mediated disynaptic inhibition. Analysis of presynaptic inputs to L5B populations revealed that PV5B mediated a switch from IT- to PT-predominant activity during movement. These predictions are consistent with findings associating IT and PT5B_upper_ with motor planning and PT5B_lower_ with motor execution.^18,57^

Dimensionality reduction of the simulated activity uncovered low-dimensional manifolds that captured neural dynamics during different behaviors and manipulations. Strong evidence supports the existence of low-dimensional manifolds and latent variables in M1 and their potential role in generating motor behavior.^67^

In summary, our mouse M1 microcircuit model has been validated against cell-type- and layer-specific mouse M1 *in vivo* data associated with different behaviors and experimental manipulations. The model provides a quantitative theoretical framework to integrate and interpret M1 experimental data across scales, evaluate hypotheses, and generate experimentally testable predictions. The model and analysis source code, simulation results, and experimental data are available online for future research and model extensions (see data and code availability).

### Limitations of the study

We aimed to create a comprehensive computational model of the mouse M1 microcircuit. We necessarily fell short due to lack of sufficient data on certain molecular, cellular, network, and connectivity aspects. Our model underwent several updates during the 6-year development and evaluation period, but like any neurobiological model, it requires continuous improvements as new data become available.

Cell models are precisely tuned to reproduce experimental somatic responses, but limited data are available to characterize dendritic ion channel densities. This, for example, affects the integration of distal synaptic inputs in L5 neurons.^68^ In terms of the influence of NA, here we focused on one effect on the PT cell type, neglecting the wide-ranging effects of this and other neuromodulators such as dopamine and acetylcholine.^69-71^ For example, NA has been shown to affect not only cortical pyramidal neurons but also inhibitory cells.^72^ We also did not include the effect of neuromodulators on second messenger cascades.^73-75^

The model does not include inhibitory cell types of the 5HT3aR class, such as interneurons expressing vasoactive intestinal polypeptides (VIP+) and cholecystokinin (CCK+) and neurogliaform cells.^76^ The properties and role of these cell types were historically less well characterized,^77^ but we now acknowledge their relevance and include them in our latest cortical models,^40^ including a new M1 version under development. Although we adapted the morphology and physiology of IT cells based on their layer, we omitted cellular diversity within each model population, i.e., we used identical morphologies and channel parameters. This contrasts with other models that vary channel conductances and morphologies.^36^

We are limited not only by lack of precise data for parameter determination but also by computational constraints. Often, network simulations use point neurons to avoid the computational load of multicompartment neurons but at the expense of accuracy.^78-80^ Here, we compromised by using relatively small multicompartment models for most populations, with the exception of two key L5 populations. Even with these compromises, optimizing and exploring our large network model required millions of HPC core hours.

Due to the nature of our circuit mapping methods,^6,15,30^ our model used local excitatory connectivity based primarily on postsynaptic cell type and presynaptic locations. Our model’s normalized cortical-depth-dependent connectivity provided greater resolution than traditional layer-based wiring. However, it did not provide cell-to-cell resolution and still contained boundaries where connection density changed sharply. This limitation in the spatial resolution of the experimental measurements was carried over to the model. The model predicts that these sharp connectivity changes translate into sharp differences in activity. However, if such sharp connectivity changes are present *in vivo*, other factors not accounted for in the model (e.g., compensatory inhibitory mechanisms) may balance the activity of neurons. In future model versions, this could be improved by using updated connectivity data with higher spatial resolution and/or by fitting discretely binned experimental data to functions of cortical depth, resulting in smoother connectivity profiles.

The model employs long-range input sources with constant average firing rates, which are unlikely to capture the spatiotemporal diversity of M1 *in vivo* responses. This could be improved in future versions by incorporating time-varying long-range inputs based on experimental recordings. Despite their fixed average firing rates, current long-range inputs generate Poisson-distributed spike times that vary over time and across regions. Furthermore, the diversity of long-range projections included in the model, as well as their layer and cell type specificity, surpasses that of any previous motor cortex circuit model.

Although we included example voltage traces similar to those recorded experimentally, this was simply to illustrate the multiple model scales and that the spiking activity was based on the underlying neuronal membrane voltages. However, closely reproducing the voltage traces of different cell types *in vivo* was out of the scope of this study.

We recognize that the biophysical origin of firing rate and LFP responses *in vivo* and the model may differ. To address this uncertainty, the model predictions about the biophysical mechanisms underlying these responses can be investigated experimentally, and the model can then be refined accordingly. We emphasize that this study constitutes the first iteration of a framework that should be iteratively updated based on new data and experimental validation to enhance the model’s predictive power over time.

### M1 cellular and circuit mechanisms associated with quiet and movement behaviors

A key question in motor system research is how motor cortex activity gets dissociated from muscle movement during motor planning or mental imagery and is then shifted to produce commands for action.^81-83^ One hypothesis has been that this planning-to-execution switch could be triggered by NA neuro-modulation.^23^ Downregulation of *I*_h_, mediated by NA and other neuromodulatory factors, has been shown to increase PT activity due to enhanced temporal and spatial synaptic integration of excitatory postsynaptic potentials (EPSPs).^23,68^ This effect is observed primarily in PT cells given their higher concentration of HCN channels compared to IT cells.^23,24^

An additional hypothesis to explain differential planning and movement outputs posits that the shift results from activation of different cell populations in L5, mediated by distinct local and long-range inputs. Evidence suggests that inputs arising from MTh carrying cerebellar signals selectively target M1 populations.^15^ These inputs are involved in movement initiation^28^ and contribute to the successful execution of dexterous movement.^27^ Using *in vivo* electrophysiology and optogenetic perturbations in mouse anterolateral motor cortex, Li et al.^18^ found evidence suggesting that preparatory activity in IT neurons is converted into a movement command in PT neurons. Additional evidence supporting this hypothesis was provided by a study that demonstrated the existence of distinct transcriptomically-identified PT subtypes in the upper and lower regions of layer 5B. This study revealed that the PT5B_upper_ subtype projected to the thalamus and was responsible for generating early preparatory activity. In contrast, the PT5B_lower_ subtpye projected to the medulla and generated motor commands.

These two hypotheses are not incompatible, and indeed our simulations suggest that both mechanisms may coexist and be required for movement-related activity (Figure 6). NA modulation and MTh input by themselves produced an increase in the overall activity of PT5B, although primarily in the preparatory activity-related PT5B_upper_ population; however, both mechanisms were required to activate the PT5B_lower_ population associated with motor commands.^57^ Analyzing synaptic drive to PT5B identified the upper and lower subpopulations based on their distinct presynaptic input patterns (Figure 7). Therefore, the model predicts that the transition to motor execution (self-paced, voluntary movement) might require both the neuromodulatory prepared state and circuit-level routing of inputs. Different types of behaviors and contexts (e.g., goal-directed behaviors with sensory feedback) may involve driving input from other populations or regions, such as supragranular layers or somatosensory cortex.^15,28,84,85^ We note that in our model and *in vivo* experiments^26^ the quiet state does not correspond to a preparatory state, as it lacks short-term memory, delays, and other preparatory components. Therefore, it remains an open question whether the previous task-related findings^18,57^ on the role of PT5B_lower_ and PT5B_upper_ generalize to our self-paced voluntary movement results.

### Simulating experimental manipulations: MTh inactivation and NA-R blocking

We gained insight into the circuitry and mechanisms governing M1 dynamics by attempting to reproduce extreme conditions posed by experimental manipulations. After MTh inactivation in our baseline model, we observed higher firing rates than *in vivo*, particularly for the quiet state. MTh inactivation is likely to affect other afferent regions of M1, such as cM1 and S2, either directly (e.g., VL → S2 → M1) and/or indirectly via recurrent interareal projections (e.g., VL → M1 → S2 → M1).^30^ We improved the model by reducing activity in the cM1 and S2 model regions during MTh inactivation, which resulted in a closer match to *in vivo* rates (Figure 5). Other factors may also explain the observed discrepancies, such as movement-related activity depending on changes in spiking patterns and not just amplitude (e.g., bursts or oscillatory activity) or being driven not only by VL but by other long-range inputs, as suggested by recent findings.^28^

Similarly, to simulate the NA-R-block condition, we modified the model to increase not only PT *I*_h_ but also K^+^ conductance in all pyramidal neurons, as suggested by multiple studies.^63,64^ This resulted in a closer match between model and experiment. Alternative explanations for the initial differences observed include selective NA modulation of inhibitory synapses^72^ and interactions with other neuromodulators such as acetylcholine.^86^ Our model can be used to explore these molecular- and cellular-level mechanisms and gain insights into their circuit-level effects.

### IT and PT disynaptic inhibition via shared L5 interneuron pools

L5B IT and PT neurons exhibited an inverse response to increased NA inputs: IT rates decreased, while PT rates increased (Figure 6). Analysis of cell-type-specific input drive to L5B populations revealed that the main excitatory driver of PV5B switches from L5 IT to PT during movement and that PV5B acts to suppress the less predominant L5 excitatory population (Figure 7). Our model predicts the computation performed by this particular subcircuit, namely, a switching mechanism between IT-and PT-predominant output modes; mutual inhibition ensures that only one of them responds strongly at a time. This is consistent with their suggested complementary roles in motor preparation vs. execution.^18^ It is also in line with the finding of shared interneuron pools in L5 IT and PT neurons mediating disynaptic inhibition,^87^ which contrasts with the private (nonshared) interneuron pools identified for PT and CT neurons.^29^ Additional support comes from *in vivo* rat results showing that PV neurons were predominantly recruited during motor execution and can shape motor commands through balanced or recurrent inhibition of output-related pyramidal neurons (PT) while suppressing pyramidal neurons (IT) associated with other functions such as hold-related activity.^88^

### Emergence of behavior-dependent physiological oscillations

Strong oscillations were observed in the delta and beta/gamma ranges with specific frequency dependence on cell class, cortical depth, and behavioral state. Simulations reproduced the decrease in delta and increase in gamma power of M1 L5 LFPs during movement observed in the *in vivo* dataset^26^ and previously reported in mouse vibrissal M1 during whisking.^61^ The model predicted the cell-type-specific origins of behavior-related changes in LFP.

Strong LFP beta and gamma oscillations are characteristic of motor cortex activity in both rodents^89,90^ and primates^91,92^ and have been found to enhance signal transmission in mouse neocortex.^93^ Both beta and gamma oscillations may play a role in information coding during preparation and execution of movements.^90,94^

### Low-dimensional latent dynamics

Low-dimensional representations of network activity clustered according to different levels of NA and VL inputs related to behavior and experimental manipulations. Attempts to reconstruct high-dimensional activity from low-dimensional embedding were remarkably successful (66% and 97% correlation for cell and population firing rates), suggesting that latent dynamics may underlie model neuronal activity despite not being built in. The similarity of movement dynamics after NA- or VL-related lesions to the control quiet state dynamics is consistent with behavioral deficits associated with these lesions.^26,28,62^

Applying dimensionality reduction to large-scale cortical models, where we have access to the activity of all neurons, can serve to (1) link network dynamics to behavior, manipulations, and disease, (2) further validate and improve the model by comparing it with experimental low-dimensional dynamics, and (3) characterize the relation between low-dimensional latent dynamics and the activity of specific layers and cell types at various recording scales, including membrane voltages, spikes, LFPs, and electroencephalogram (EEG). This may lead to a better understanding of how the brain circuits generate motor behavior.^60^,^95^

### Implications for experimental research and therapeutics

Our model integrates previously isolated experimental data at multiple scales into a unified simulation that can be progressively extended as new data become available. This *in silico* testbed can be systematically probed to study circuit dynamics and biophysical mechanisms with a level of resolution and precision not available experimentally. Unraveling the nonintuitive multiscale interactions occurring in M1 circuits^96^ can help us understand disease mechanisms and develop pharmacological and neurostimulation treatments.^22,35,97-101^

## STAR★METHODS

### RESOURCE AVAILABILITY

#### Lead contact

Further information and requests for resources and reagents should be directed to and will be fulfilled by the lead contact, Salvador Dura-Bernal (salvador.dura-bernal@downstate.edu).

#### Materials availability

This study did not generate new materials. Links to the model source code, analysis code, experimental data and simulation data are available in the data and code availability subsection below.

#### Data and code availability

The model source code, analysis source code, experimental data to constrain and validate the model, and simulation output data used in this study are available at https://github.com/suny-downstate-medical-center/M1_NetPyNE_CellReports_2023 and Zenodo Data: https://doi.org/10.5281/zenodo.7991991. The model source code is also available at http://modeldb.yale.edu/260015. The model is defined using the NetPyNE specifications, a JSON-based human-readable language, which can be exported to the SONATA and NeuroML standardized formats.

We have also made the M1 model available on the Open Source Brain (OSB) online platform, which enables users to explore the model via the NetPyNE Graphical User Interface (GUI): https://v2.opensourcebrain.org/repositories/60. This allows users to explore all components of the network (including cell properties, synaptic properties, populations, connectivity, stimulation, etc), run simulations and visualize and analyze the network connectivity and simulation results (membrane voltages, spike raster plots, LFPs, etc.). The OSB infrastructure currently offers running simulations on up to 4 cores. However, it is being extended to provide access to the Neuroscience Gateway (NSG) and other public supercomputers directly from the NetPyNE GUI. Therefore, although at the time of publication only smaller-scale versions of the M1 model can be simulated, it will soon be possible to run the full-scale M1 model online via OSB and the NetPyNE GUI. OSB also provides users with a permanent workspace and file system storage. The link above includes step-by-step instructions on how to explore the M1 model using the NetPyNE GUI. We will update these instructions as the OSB and NetPyNE GUI tools evolve.

Any additional information required to simulate the model or analyze the data reported in this paper is available from the lead contact upon request.

### METHOD DETAILS

This section describes model development with data provenance, and major features of the final model, as well as the analysis and experimental methods. The full documentation of the final model and analyses is the source code itself (see resource availability above).

#### Morphology and physiology of neuron classes

Seven excitatory pyramidal cell and two interneuron cell models were employed in the network. Their morphology and physiological responses are summarized in Figure 1A,B,C and S1B. In previous work we developed layer 5B PT corticospinal cell and L5 IT corticostriatal cell models that reproduced *in vitro* electrophysiological responses to somatic current injections, including sub- and super-threshold voltage trajectories and f-I curves.^47,102^ To achieve this, we optimized the parameters of the Hodgkin-Huxley neuron model ionic channels – Na, Kdr, Ka, Kd, HCN, CaL, CaN, KCa – within a range of values constrained by the literature. The corticospinal and corticostriatal cell model morphologies had 706 and 325 compartments, respectively, digitally reconstructed from 3D microscopy images. Morphologies are available via NeuroMorpho.org ^103^(archive name “Suter_Shepherd”). For the current simulations, we further improved the PT model by 1) increasing the concentration of Ca^2+^ channels (“hot zones”) between the nexus and apical tuft, following parameters published in^104^; 2) lowering dendritic Na+ channel density in order to increase the threshold required to elicit dendritic spikes, which then required adapting the axon sodium conductance and axial resistance to maintain a similar f-I curve; 3) replacing the HCN channel model and distribution with a more recent implementation.^105^ Although the Kole^106^ implementation is more in line with the kinetics of HCN channels, PT neurons with the new HCN channel^105^ reproduced some experimental observations that our PT cells with the previous implementation^106^ could not. These include 1) the change from excitatory to inhibitory effect in response to synaptic inputs of increasing strength,^107^ and 2) phase responses to oscillatory inputs.^108^ This was achieved by including a shunting current proportional to *I*_h_. We tuned the HCN parameters (*lk* and *v*_rev_lk_) and passive parameters to reproduce the reported change from excitation to inhibition as a function of synaptic strength, and the experimental f-I curve.

The network model includes five other excitatory cell classes: layer 2/3, layer 4, layer 5B and layer 6 IT neurons, and layer 6 CT neurons. Since our focus was on the role of L5 neurons, other cell classes were implemented using simpler models as a trade-off to enable running a larger number of exploratory network simulations. Previously, we had optimized 6-compartment neuron models to reproduce somatic current clamp recordings from two IT cells in layers 5A and 5B. The layer 5A cell had a lower f-I slope (77 Hz/nA) and higher rheobase (250 nA) than that in layer 5B (98 Hz/nA and 100 nA). Based on our own and published data, we found two broad IT categories based on projection and intrinsic properties: corticocortical IT cells found in upper layers 2/3 and 4 which exhibited a lower f-I slope (~72 Hz/nA) and higher rheobase (~281 pA) than IT corticostriatal cells in deeper layers 5A, 5B and 6 (~96 Hz/nA and ~106 pA).^43,47,109^ The rheobase and slope (69 Hz/nA and 298 pA) of the CT neurons f-I curve was closer to that of corticocortical neurons.^109^ We therefore employed the layer 5A IT model for layers 2/3 and 4 IT neurons and layer 6 CT neurons, and the layer 5B IT model for layers 5A, 5B and 6 IT neurons. We further adapted cell models by modifying their apical dendrite length to match the average cortical depth of the layer, thus introducing small variations in the firing responses of neurons across layers.

We implemented models for two major classes of GABAergic interneurons^24,110,111^: parvalbumin-expressing (PV) fast-spiking and somatostatin-expressing (SOM) low-threshold spiking neurons. We employed existing simplified 3-compartment (soma, axon, dendrite) models^112^ and increased their dendritic length to better match the average f-I slope and rheobase experimental values of cortical basket (for PV) and Martinotti (for SOM) cells (Neuroelectro online database^113^).

#### Microcircuit composition: Neuron locations, densities and ratios

We modeled a cylindric volume of the mouse M1 cortical microcircuit with a diameter of 300 *μm* and a height (cortical depth) of 1350 *μm* at full neuronal density for a total of 10,073 neurons (Figure 1). Cylinder diameter was chosen to approximately match the horizontal dendritic span of a corticospinal neuron located at the center, consistent with the approach used in the Blue Brain Project model of the rat S1 microcircuit.^36^ Mouse cortical depth and boundaries for layers 2/3, 4, 5A, 5B and 6 were based on our published experimental data.^6,17,43^ Although traditionally M1 has been considered an agranular area lacking layer 4, we recently identified M1 pyramidal neurons with the expected prototypical physiological, morphological and wiring properties of layer 4 neurons^43^ (see also^24,44,45^), and therefore incorporated this layer in the model.

Cell classes present in each layer were determined based on mouse M1 studies.^6,43,47,77,109^ IT cell populations were present in all layers, whereas the PT cell population was confined to layer 5B, and the CT cell population only occupied layer 6. SOM and PV interneuron populations were distributed in each layer. Neuronal densities (neurons per *mm*^3^) for each layer (Figure 1C) were taken from a histological and imaging study of mouse agranular cortex.^114^ The proportion of excitatory to inhibitory neurons per layer was obtained from mouse S1 data^115^ (Figure S1A). The proportion of IT to PT and IT to CT cells in layers 5B and 6, respectively, were both estimated as 1:1.^29,47^ The ratio of PV to SOM neurons per layer was estimated as 2:1 based on mouse M1 and S1 studies^116,117^ (Figure S1A). Since data for M1 layer 4 was not available, interneuron populations labeled PV5A and SOM5A occupy both layers 4 and 5A. The number of cells for each population was calculated based on the modeled cylinder dimensions, layer boundaries and neuronal proportions and densities per layer.

#### Local connectivity

We calculated local connectivity between M1 neurons (Figures 1C and S1C) by combining data from multiple studies. Data on excitatory inputs to excitatory neurons (IT, PT and CT) was primarily derived from mapping studies using whole-cell recording, glutamate uncaging-based laser-scanning photostimulation (LSPS) and subcellular channelrhodopsin-2-assisted circuit mapping (sCRACM) analysis.^6,17,29,43^ Connectivity data was postsynaptic cell class-specific and employed normalized cortical depth (NCD) instead of layers as the primary reference system. Unlike layer definitions which can be interpreted differently between studies, NCD provides a well-defined, consistent and continuous reference system, depending only on two readily-identifiable landmarks: pia (NCD = 0) and white matter (NCD = 1). Incorporating NCD-based connectivity into our model allowed us to capture wiring patterns down to a 100 *μm* spatial resolution, well beyond traditional layer-based cortical models. M1 connectivity varied systematically within layers. For example, the strength of inputs from layer 2/3 to L5B corticospinal cells depends significantly on cell soma depth, with upper neurons receiving much stronger input.^6^

Connection strength thus depended on presynaptic NCD and postsynaptic NCD and cell class. For postsynaptic IT neurons with NCD ranging from 0.1 to 0.37 (layers 2/3 and 4) and 0.8 to 1.0 (layer 6) we determined connection strengths based on data from^17^ with cortical depth resolution of 140 *μm*-resolution. For postsynaptic IT and PT neurons with NCD between 0.37 and 0.8 (layers 5A and 5B) we employed connectivity strength data from^6^ with cortical depth resolution of 100 *μm*. For postsynaptic CT neurons in layer 6 we used the same connection strengths as for layer 6 IT cells,^17^ but reduced to 62% of original values, following published data on the circuitry of M1 CT neurons.^29,118^ Our data^29^ also suggested that connection strength from layer 4 to layer 2/3 IT cells was similar to that measured in S1, so for these projections we employed values from Lefort’s S1 connectivity strength matrix.^115^ Experimentally, these connections were found to be four times stronger than in the opposite direction – from layer 2/3 to layer 4 – so we decreased the latter in the model to match this ratio.

Following previous publications,^14,115^ we defined connection strength (*s*_*con*_, in mV) between two populations, as the product of their probability of connection (*p*_*con*_) and the unitary connection somatic EPSP amplitude in mV (*v*_*con*_), i.e. *s*_*con*_ = *p*_*con*_ × *v*_*con*_. We employed this equivalence to disentangle the connection *s*_*con*_ values provided by the above LSPS studies into *p*_*con*_ and *v*_*con*_ values that we could use to implement the model. First, we rescaled the LSPS raw current values in pA^6,17,29,43^ to match *s*_*con*_ data from a paired recording study of mouse M1 L5 excitatory circuits.^14^ Next, we calculated the M1 NCD-based *v*_*con*_ matrix by interpolating a layerwise unitary connection EPSP amplitude matrix of mouse S1,^115^ and thresholding values between 0.3 and 1.0 mV. Finally, we calculated the probability of connection matrix as *p*_*con*_ = *s*_*con*_*/v*_*con*_.

To implement *v*_*con*_ values in the model we calculated the required NEURON connection weight of an excitatory synaptic input to generate a somatic EPSP of 0.5 mV at each neuron segment. This allowed us to calculate a scaling factor for each segment that converted *v*_*con*_ values into NEURON weights, such that the somatic EPSP response to a unitary connection input was independent of synaptic location – also known as synaptic democracy.^119,120^ Evidence from CA1 pyramidal neurons shows that synaptic conductances increased with distance from soma, to normalize somatic EPSP amplitude of inputs within 300 *μm* of soma.^121^ Although this effect is not evident in L5 cortical pyramidal neurons,^122^ there is some evidence that active conductances in cortical neuron dendrites can normalize the efficacy of distal synapses.^120^ Scaling factors calculated for PT cell apical tufts were very high and resulted in overexcitability in network simulations where each cell can receive hundreds of inputs that interact nonlinearly.^123,124^ We, therefore, thresholded all scaling factors to a maximum of 4.0. For morphologically detailed cells (layer 5A IT and layer 5B PT), the number of synaptic contacts per unitary connection (or simply, synapses per connection) was set to five, an estimated average consistent with the limited mouse M1 data^125^ and rat S1 studies.^36,126^ Individual synaptic weights were calculated by dividing the unitary connection weight (*v*_*con*_) by the number of synapses per connection. Although the method does not account for nonlinear summation effects,^123^ it provides a reasonable approximation and enables employing a more realistic number and spatial distribution of synapses, which may be key for dendritic computations.^127^ For the remaining cell models, all with six compartments or less, a single synapse per connection was used.

For excitatory inputs to inhibitory cell types (PV and SOM) we started with the same values as for IT cell types but adapted these based on the specific connectivity patterns reported for mouse M1 interneurons^29,87^ (Figure S1C). Following the layer-based description in these studies, we employed three major subdivisions: layer 2/3 (NCD 0.12 to 0.31), layers 4, 5A and 5B (NCD 0.31 to 0.77) and layer 6 (NCD 0.77 to 1.0). We increased the probability of layer 2/3 excitatory connections to layers 4, 5A and 5B SOM cells by 50% and decreased that to PV cells by 50%.^87^ We implemented the opposite pattern for excitatory connections arising from layer 4.5A,5B IT cells such that PV interneurons received stronger intralaminar inputs than SOM cells.^87^ The model also accounts for layer 6 CT neurons generating relatively more inhibition than IT neurons.^29,118^ Inhibitory connections from interneurons (PV and SOM) to other cell types were limited to neurons in the same layer,^116^ with layers 4,5Aand 5B combined into a single layer.^77^ Probability of connection decayed exponentially with the distance between the pre- and post-synaptic cell bodies with a length constant of 100 *μm*^66,67^. We introduced a correction factor to the distance-dependent connectivity measures to avoid the *border effect*, i.e. cells near the modeled volume edges receiving less or weaker connections than those in the center.

For comparison with other models and experiments, we calculated the probability of connection matrices arranged by population (instead of NCD) for the base model network instantiation used throughout the results. (Figure S1D).

Excitatory synapses consisted of colocalized AMPA (rise, decay τ: 0.05, 5.3 ms) and NMDA (rise, decay τ: 15, 150 ms) receptors, both with reversal potential of 0 mV. The ratio of NMDA to AMPA receptors was 1.0,^128^ meaning their weights were each set to 50% of the connection weight. NMDA conductance was scaled by 1/(1 + 0.28·*Mg*·exp(−0.062·*V*)); Mg = 1mM.^129^ Inhibitory synapses from SOM to excitatory neurons consisted of a slow *GABA*_*A*_ receptor (rise, decay τ: 2, 100 ms)^130,131^ and *GABAs* receptor, in a 90%–10% proportion; synapses from SOM to inhibitory neurons only included the slow *GABA*_*A*_ receptor; and synapses from PV to other neurons consisted of a fast *GABA*_*A*_ receptor (rise, decay τ: 0.07, 18.2). The reversal potential was −80 mV for *GABA*_*A*_ and −95 mV for *GABA*_*B*_. We note that some synaptic parameter values may need updating based on more recent data, such as the 100 ms day time used in the slow *GABA*_*A*_ receptor of SOM cells. The *GABA*_*B*_ synapse was modeled using second messenger connectivity to a G protein-coupled inwardly-rectifying potassium channel (GIRK).^132^ The remaining synapses were modeled with a double-exponential mechanism.

Connection delays were estimated as 2 ms plus a variable delay depending on the distance between the pre- and postsynaptic cell bodies assuming a propagation speed of 0.5 m/s.

#### Long-range input connectivity

We added long-range input connections from seven regions that are known to project to M1: thalamic posterior nucleus (PO), ventrolateral thalamus (VL), primary somatosensory cortex (S1), secondary somatosensory cortex (S2), contralateral primary motor cortex (cM1), secondary motor cortex (M2) and orbital cortex (OC). We note that VL constitutes the largest nuclei of the motor thalamus (MTh) so, in the context of the model, these terms are equivalent. Each region consisted of a population of 1000^126,133^ spike-generators (NEURON VecStims) that generated independent random Poisson spike trains with uniform distributed rates between 0 and 2.5 Hz or 0 and 5 Hz^52,53^ for spontaneous firing; 0 and 10 Hz^88,134^ when simulating increased input from a region. Previous studies provided a measure of normalized input strength from these regions as a function of postsynaptic cell type and layer or NCD. Broadly, PO,^15,29,43^S1^58,85^ and S2^30^ projected strongly to IT cells in layers 2/3 and 5A (PO also to layer 4); VL projected strongly to PT cells and to layer 4 IT cells^15,29,43^; cM1 and M2 projected strongly to IT and PT cells in layers 5B and 6^15^; and OC projected strongly to layer 6 CT and IT cells.^15^ We implemented these relations by estimating the maximum number of synaptic inputs from each region and multiplying that value by the normalized input strength for each postsynaptic cell type and NCD range. This resulted in a convergence value – average number of synaptic inputs to each postsynaptic cell – for each projection (Figure S1D). We fixed all long-range input connection weights (unitary connection somatic EPSP amplitude) to 0.5 mV, as an approximation consistent with rat and mouse S1 data.^125^,^133^

To estimate the maximum number of synaptic inputs from each long-range input region, we made a number of assumptions based on the limited data available (Figures S1E and S1C). First, we estimated the average number of synaptic contacts per cell as 8234 by rescaling rat S1 data^135^ based on our own observations for PT cells^47^ and contrasting with related studies^136,137^; we assumed the same value for all cell types so we could use convergence to approximate long-range input strength. We assumed 80% of synaptic inputs were excitatory vs. 20% inhibitory^36,137^; out of the excitatory inputs, 80% were long-range vs. 20% local^36,138^; and of the inhibitory inputs, 30% were long-range vs. 70% local.^138^ Finally, we estimated the percentage of long-range synaptic inputs arriving from each region based on mouse brain mesoscale connectivity data^139^ and other studies.^44,126,135,140,141^

Experimental evidence demonstrates the location of synapses along dendritic trees follows very specific patterns of organization that depend on the brain region, cell type and cortical depth^30,41^; these are likely to result in important functional effects.^123,142,143^ We employed sCRACM data to estimate the synaptic density along the dendritic arbor – 1D radial axis – for inputs from PO, VL, M2 and OC to layers 2/3, 5A, 5B and 6 IT and CT cell,^15^ and from layer 2/3 IT, VL, S1, S2, cM1 and M2 to PT neurons^30^ (Figure S1E). To approximate radial synaptic density we divided the sCRACM map amplitudes by the dendritic length at each grid location, and averaged across rows. Once all network connections had been generated, synaptic locations were automatically calculated for each cell based on its morphology and the pre- and postsynaptic cell-type-specific radial synaptic density function (Figure S1F). Synaptic inputs from PV to excitatory cells were located perisomatically (50 *μm* around soma); SOM inputs targeted apical dendrites of excitatory neurons^77,116^; and all inputs to PV and SOM cells targeted apical dendrites. For projections where no synaptic distribution data was available – IT/CT, S1, S2 and cM1 to IT/CT cells – we assumed a uniform dendritic length distribution.

#### Model parameter optimization

Although we followed a systematic data-driven approach to build the model, the full experimental multiscale dataset required to build a detailed model of mouse M1 circuits was not available. Consequently, we had to include some data from different species and cortical regions. Furthermore, data was obtained from many different experiments and using different recording methods. It is therefore not surprising that in order to obtain physiologically constrained firing rates across all model populations, it was necessary to fine-tune some of the connectivity parameters.

We built the model by progressively adding features and fixing bugs and issues over time. Examples include adding an improved HCN channel model to PT cells or updating connectivity from the thalamus to layer 4 based on a more recent publication.^43^ Each version of the model was labeled with a new identifier. The version used for this publication was v56, indicating that there were 56 sets of updates or fixes to the model. Each model version typically required running several simulations to test and debug it.

The final step involved optimizing some of the long-range input and connectivity parameters to obtain physiologically realistic firing rates in all populations. For this purpose, we used the iterative grid search method, which we explored a small subset of parameter values and selected the best results for the next iteration. In this next iteration we explored a refined subset of parameter values. To evaluate the results we employed two different values of *I*_h_ conductance (25% and 100% of baseline). We aimed to satisfy two criteria: 1) physiologically reasonable firing rates for all populations with baseline *I*_h_ (approximately 0.1–10 Hz for excitatory populations and 5–40 Hz for inhibitory), and low *I*_h_ (approximately 0.1–30 Hz for excitatory populations and 5–80 Hz for inhibitory); and 2) an increase in PT5B firing rates from high to low *I*_h_.

The optimized parameters included 1) the average firing rate of the 7 long-range inputs (TPO, TVL, S1, S2, cM1, M2 and OC), which ranged from 2 to 6 Hz in discrete intervals; and 2) a scaling factor for the connectivity weights of all E-E connections, which ranged from 0.5 to 1.0 in 0.1 intervals; and 3) a scaling factor for the connectivity weights of all I-E and I-I connections separate for 3 groups of layers i) L2/3 and L4, ii) L5A and L5B and ii) L6, i.e. a total of 6 parameters, each of which ranged from 0.8, 1.0 and 1.2. These scaling factors affected thousands of individual synaptic connections for that particular projection type and layer. The total number of simulations during this final iterative optimization process was approximately 1000.

We evaluated the grid search simulation results and selected the solution that best matched the criteria described above. We note that we did not automate the selection process or use as a target any of the specific experimental data included in the statistical comparison in Figure 2 (these publications were identified later for validation purposes). Our optimization criteria were largely based on rodent somatosensory cortex data and personal communication with the experimental coauthors.

#### Modeling and simulation tools

The model was developed using the parallel NEURON (http://neuron.yale.edu)^144,145^ simulation engine the and NetPyNE (http://netpyne.org)^42^ multiscale modeling tool. NetPyNE is a Python package to facilitate the development, optimization, parallel simulation and analysis of biological neuronal networks using the NEURON simulator. NetPyNE emphasizes the incorporation of multiscale anatomical and physiological data at varying levels of detail. It converts a set of simple, standardized high-level specifications in a declarative format into a NEURON model. This high-level language enables, for example, defining connectivity as a function of NCD, and distributing synapses across neurons based on normalized synaptic density maps. NetPyNE facilitates running parallel simulations by taking care of distributing the workload and gathering data across computing nodes, and automates the submission of batches of simulations for parameter optimization and exploration. It also provides a powerful set of analysis methods so the user can plot spike raster plots, LFP power spectra, information transfer measures, connectivity matrices, or intrinsic time-varying variables (eg. voltage) of any subset of cells. To facilitate data sharing, the package saves and loads the specifications, network, and simulation results using common file formats (Pickle, MATLAB, JSON or HDF5), and can convert to and from NeuroML^146,147^ and SONATA,^148^ standard data formats for exchanging models in computational neuroscience. Simulations were run on Google Cloud, XSEDE supercomputers Comet and Stampede (using the Neuroscience Gateway (NSG) and our own resource allocations), and on the Human Brain Project (HBP)/EBRAINS ICEI Fenix Infrastructure.

#### Parameter exploration/optimization

We ran batch simulations to explore and optimize parameters using NetPyNE’s automated built-in methods. We specified the range of parameters and parameter values to explore and the tool automatically submitted the jobs in multicore machines (using NEURON’s bulletin board) or HPCs (using SLURM). We primarily employed the SLURM workload manager on San Diego Supercomputer Center’s (SDSC) Comet supercomputer and on Google Cloud Platform. We customized pre-defined batch simulation setups for different environments.

#### Local field potentials

The local field potential (LFP) signal at each simulated electrode was calculated using the “line source approximation”,^149-151^ which is based on the sum of the membrane current source generated at each cell segment divided by the distance between the segment and the electrode. The calculation assumes that the electric conductivity (sigma = 0.3 mS/mm) and permittivity of the extracellular medium are constant everywhere and do not depend on frequency. The above method is built into the NetPyNE tool, which we used to simulate the LFP signals obtained from extracellular electrodes located at multiple depths within the cortical network.

To compute the contribution of each population to the L5 LFP recorded signal (Figures 4E and 4F), we employed the same approach above but used exclusively the recorded transmembrane currents from a specific population.

#### Systematic exploration of MTh and NA input levels on L5B firing rates

To generate the heatmap of experimental firing rates as a function of MTh and NA inputs levels (Figure 7A, top), we combined experimental results from the control, MTh inactivation and NA-R block conditions, resulting in six data points, and extrapolated the remaining three data points. For the model heatmap (Figure 7A, bottom) results correspond to the original model version (without the modifications proposed for MTh inactivation and NA-block conditions), and we ran additional simulations for each of the 25 parameter combinations of MTh and NA values shown in the figure.

#### Low-dimensional representation of network dynamics

To produce a low-dimensional representation of the network activity, we considered two approaches to generate the starting high-dimensional space. The first, focused on the cell level, was to randomly select 1000 neurons from the entire spiking network and compute the average firing rate at 25-ms time steps, such that each time step corresponds to a 1000-dimensional vector. The second, focused on the population level, was to compute the average firing rate at 10-ms time steps for each of the 16 populations (we split PT5B into upper and lower subpopulation due to their distinct properties). The population-level approach is feasible for in silico simulations where we have access to all the different cell types across layers in a reproducible manner. However, the cell-level approach is most commonly used currently due to the limited number of identified neurons that can be simultaneously recorded from.^59^ Therefore, although we employ both approaches for comparison in Figure 3, for the rest of the figures we selected the cell-level approach. We employed 2 s of simulation for each behavior and/or condition. We pooled data from five simulations with different input randomization seeds, which mimic different trials in behavioral experiments.

Different techniques can be used to reduce the dimensionality of the high-dimensional representation of network activity. The particular technique selected will depend on the interpretability of the results obtained, the quality of the visualization displayed in the low-dimensional space, and the ability to reconstruct the original activity, among other factors. In our case, since the network is settled on a few fixed operational points (quiet and movement behavioral states), without a clear evolving temporal pattern, we prioritized visualizations supporting the conclusions drawn from quantitative measures such as changes in firing rates. In this regard, the uniform manifold approximation and projection (UMAP) technique outperformed principal component analysis (PCA) and t-distributed stochastic neighbor embedding (t-SNE).

We, therefore, employed UMAP to generate 3D representations from the 1000- or 16-dimensional data describing the network spiking activity over time. In brief, UMAP is a nonlinear dimension reduction method that estimates manifolds and generates a projection that preserves the topological structure of the data. Trajectories were smoothed with the *csaps* Python package, based on cubic smoothing splines for univariate and multivariate n-dimensional data, using a *smooth* value equal to one, which enforces trajectories to be very close to data points. Several hyperparameters can have a significant impact on the resulting UMAP embedding: *n_components*, the dimension of the low-dimensional space, which we set to 3 for all results; *n_neighbors*, which balances local versus global structure in the data; and *min_dist*, which controls how tightly points are packed together in the low-dimensional space. We used the following hyperparameter values: *n_neighbors* = 100 (Figures 3C), 15 (Figures 3D), 100 (Figure 5), 100 (Figure 6), 100 (Figure S2), 100 (Figure S3; *mimdist* = 0.95 (Figures 3C), 0.30 (Figures 3D), 0.95 (Figure 5), 0.5 (Figure 6), 0.95 (Figure S2), 0.95 (Figure S3).

To help interpret the low-dimensional representation we employed the Silhouette method,^152^ which provides a measure of how close a data point is to all other points within the same class/cluster (cohesion) compared to how close it is to all points in another cluster (separation). The mean Silhouette value across all points within a cluster provides a measure of how dense and well separated the cluster is, called the Silhouette Coefficient. Its value ranges from −1 to +1, where high values indicate highly dense and well separated clusters, −1 indicates incorrect clustering, and values around 0 indicate overlapping clusters.

#### Analysis of synaptic drive from presynaptic inputs

We analyzed the synaptic inputs driving different populations to better understand changes in firing patterns across different states (e.g., quiet vs. movement) and neural populations (e.g., IT5B vs. PT5B). To do this we estimated the input strength from each presynaptic population to the target postsynaptic population. Note that to estimate synaptic drive we took advantage of the fact that the synaptic strength between two neurons is defined in the model as the somatic unitary postsynaptic potential (uPSP), in mV. Our modeling tool internally estimates the required peak synaptic conductance in nS to approximate the somatic uPSP, making all synapses similarly efficient independent of dendritic location (synaptic democracy). Therefore, we can estimate the synaptic drive between two neurons by multiplying the synaptic strength (uPSPs in mV) by the firing rate of the presynaptic cell, and summing across all synaptic contacts between the two cells. To get the total synaptic drive to each postsynaptic cell, we estimated the synaptic drive from each presynaptic cell and summed it across the presynaptic population. The last step was to average across all cells of the target postsynaptic population. The final equation for estimated synaptic drive between two populations is shown below (Equation 1):

(Equation 1) $$S=\frac{1}{N_{post}}\sum_{i=0}^{N_{post}}\sum_{j=0}^{N_{pre}^{i}}r_{j}\cdot w_{i,j}$$

where, S is the average estimated synaptic drive between a presynaptic and a postsynaptic population, $N_{post}$ is the number of cells in the postsynaptic population, $N_{pre}^{i}$ is the number of cells in the presynaptic population that project to postsynaptic cell i, $r_{j}$ is the average firing rate of presynaptic cell j (in spikes/sec), and $w_{i,j}$ is the synaptic strength between presynaptic cell j and postsynaptic cell i (in mV, as it represents somatic uPSP).

For visualization purposes, in Figure 7 we omitted the inhibitory long-range inputs since they were generally an order of magnitude smaller than the corresponding excitatory inputs. However, inhibitory long-range inputs were included in the calculation of the total (“Sum”) synaptic drive.

We note that this measure is just an estimate of synaptic drive as it ignores several factors that affect the PSP generation and integration, such as nonlinear dendritic transformations and spatiotemporal summation. For instance, our method will combine inhibitory inputs linearly, based on presynaptic firing rate, which might overestimate the drive of temporally close inhibitory inputs at hyperpolarized membrane potentials. Despite these limitations, our method estimates the cell-type-specific synaptic drives without the need to record and analyze thousands of synaptic currents for each postsynaptic cell, making the problem computationally feasible for the scale of our model.

In Figure 7 we used the UMAP technique to reduce the dimensionality of the estimated synaptic drive to PT5B from 16 dimensions (number of presynaptic populations) to 2 dimensions. We then employed k-means clustering to group the data points of the 2D UMAP projection. We also colored the UMAP 2D representation according to the postsynaptic cell average firing rate and normalized cortical depth. This allowed us to correlate these physiological and anatomical features with the presynaptic inputs to each cell.

#### Summary of modeling assumptions/limitations

Below we summarize some of the main modeling assumptions and limitations at each scale. See STAR Methods and discussion for details.

Subcellular:

- Dendritic ion channel densities constrained based on limited experimental data.
- NA effects limited to PT cells, omitting effects on other cell types and synapses.
- Omitted wide-ranging effects of other neuromodulators such as dopamine and acetylcholine.
- Used HCN model by Migliore^105^ although model by Kole^106^ more in line with HCN channel kinetics.

Cellular:

- Omitted inhibitory cell types of the 5HT3aR class, such as VIP+, cholecystokinin CCK+ and neurogliaform cells.
- Omitted cellular diversity within each model population.
- Used cell models with a relatively small number of compartments for all populations except IT5A and PT5B.

Connectivity:

- Connectivity depends on sublaminar cortical depth derived from experimental data with sharp transition boundaries.
- Implemented synaptic democracy despite limited evidence in cortical pyramidal neurons.
- Some synaptic parameters may require updating, such as the 100 ms day time used in the slow *GABA*_*A*_ receptor of SOM cells.
- Omitted synaptic plasticity.
- Long-range inputs are modeled as spike generators with average firing rates, omitting the diversity and richness of M1 *in vivo* dynamics.

Simulation output:

- Not tuned to closely reproduce the *in vivo* membrane voltage traces of different cell types.
- Sample sizes of experimental firing rate datasets were much smaller than those of model datasets.

#### Experimental procedures

Details of the experimental procedures used to obtain the data in this study were previously described in Schiemann et al.,^26^ including animals and surgery, motion index and motion pattern discrimination, and *in vivo* electrophysiology and pharmacology. The dataset on cell type-specific *in vivo* firing rates across states and conditions was collected and previously reported in the same publication. The LFP experimental data reported here was collected during that same study but only a small subset was reported in the experimental paper (ref^26^ Figure 1).

We pre-processed the experimental LFP data used in Figure 4 to remove outliers and potential artifacts. The raw LFP data consisted of 30 recordings of varying duration during head-restrained mice locomotion (at different speeds) on a cylindrical treadmill. To compare it with the simulated data, we classified the raw LFP *in vivo* into quiet and movement periods (using the same criteria as in ref^26^) and divided the signal into 4-s segments (the same duration as the model simulations). We then calculated the LFP power spectral density (PSD) using the Morlet wavelet transform method, normalized the power between 0 and 1 for each LFP segment PSD, and computed their mean normalized power across five standard frequency bands: delta (0–4 Hz), theta (4–8 Hz), alpha (8–12 Hz), beta (12–30 Hz) and gamma (30–80 Hz). This resulted in a 5-element vector for each 4-s LFP segment, representing the normalized power in each frequency band. This dataset exhibited relatively high variability: the mean coefficient of variation (CV) was 0.60 across quiet segments and 0.44 across movement segments. To explore the source of this variability, we visualized the data using UMAP dimensionality reduction and clustered them using K-Means. The number of clusters, k, was set to 2, based on the UMAP visualization. The quiet and movement states each showed a large cluster (quiet: 73% of observations; movement: 77% of observations) and a small cluster (quiet: 27% of observations; movement: 23% of observations). Noticeably, the LFP power distribution of the quiet large cluster was similar to that of the movement small cluster (similar power across all frequency bands), and the LFP power distribution of the movement large cluster was similar to that of the quiet small cluster (high gamma power). This was also reflected in the average distance between clusters in the 2D UMAP projection: QL-MS: 1.1, QS-ML = 2.4, QS-QL = 5.0, MS-ML = 6.2, QL-ML: 7.3 (where Q = quiet, M = movement, S = small cluster, L = large cluster). This suggested that the smaller clusters may result from cross-contamination of the quiet and movement data or other experimental artifacts. Alternatively, the smaller clusters may correspond to secondary behaviors with different frequency responses due to internal states, different levels of involvement in the task, or transition periods. For comparison with the model results, we therefore used the large clusters for each behavioral state, which contain approximately 75% of the data, and represent the primary or dominant LFP frequency response. As expected, the variability within each large cluster was significantly reduced compared to the full dataset (quiet CV = 0.33, movement CV = 0.32).

### QUANTIFICATION AND STATISTICAL ANALYSIS

The statistical details of our study can be found in the results main text and figure captions. For statistical analysis we used the Scipy Python package.

Firing rate statistics were always calculated starting at least 1 s after the simulation start time to allow the network to reach a steady state. To enable the statistical comparison of the results in Figure 2 we only included neurons with firing rates above 0 Hz, given that most experimental datasets^18,54,56^ already included this constrain. For the statistical comparison in the remaining sections, we included neurons with firing rates of 0 Hz, as these were available both in the experimental dataset^26^ and the model. Therefore, the quiet state mean firing rates reported in Figure 2 (which only included rates > 0 *Hz*) were higher than those in the remaining sections.

For the statistical comparisons in Figure 2 we used the Wilcoxon rank-sum test since we wanted to compare the difference in the median between two groups but the data was not normally distributed (rank-sum is a non-parametric alternative to the two-sample t test), and since the test is robust to outliers, which were present in the data.

We note that the experimental dataset represents a small sparse sample of neurons in the modeled cortical volume, resulting in a model data sample size approximately three orders of magnitude larger than that of experiments (e.g. for L5B *N*_*model*_ = 35182 vs. *N_experiment_* = 47). Therefore, validation of our model results can be understood as showing that the small dataset of experiment cell rates could have been subsampled from the larger dataset of model rates. Novel methods that record from an increasingly larger number of simultaneous neurons^153^ will enable further validation of the model results.

In the statistical comparison of experimental and model LFP data (Figure 4C), the experimental dataset included more 4-s samples for quiet states (N = 3890) than movement states (N = 2840). These were obtained from 30 recordings of different animals. To more directly quantify the change in LFP power from quiet to movement, we selected the subset of paired 4-s quiet and movement samples that occurred consecutively within the same recording. We then calculated the change in normalized LFP PSD for the resulting 160 pairs of consecutive quiet and movement samples (Figure 4D).

## Supplementary Material

Supplementary Material

## Figures and Tables

**Table:** KEY RESOURCES TABLE

REAGENT or RESOURCE	SOURCE	IDENTIFIER
Deposited data		
In Vivo Experimental Data	Schiemann et al. 2015^26^	Zenodo: https://doi.org/10.5281/zenodo.7991991
Data used to constrain M1 model	This paper	Zenodo: https://doi.org/10.5281/zenodo.7991991
M1 model output simulated data	This paper	Zenodo: https://doi.org/10.5281/zenodo.7991991
Software and algorithms		
M1 model and data analysis source code	This paper; https://github.com/suny-downstate-medical-center/M1_NetPyNE_CellReports_2023	Zenodo: https://doi.org/10.5281/zenodo.7882125
M1 model on NetPyNE GUI via OSB platform	https://v2.opensourcebrain.org/repositories/60	N/A
NetPyNE	http://netpyne.org	Zenodo: https://doi.org/10.5281/zenodo.4767870
NEURON	https://neuron.yale.edu/	N/A
Python	http://python.org	N/A

## Highlights

- Detailed model of primary motor cortex integrates experimental data across scales
- Model predicts cell-type-specific firing rates and LFPs during quiet and movement behaviors
- NA, thalamus, and PV disynaptic inhibition mediate switch between L5B PT vs. IT output
- Analyses of LFP sources, synaptic drive, and neural manifolds provide further insights

## INCLUSION AND DIVERSITY

We support inclusive, diverse, and equitable conduct of research. One or more of the authors of this paper self-identifies as an underrepresented ethnic minority in their field of research or within their geographical location. One or more of the authors of this paper self-identifies as a gender minority in their field of research. One or more of the authors of this paper received support from a program designed to increase minority representation in their field of research.
