# Supplementary material for: Multiscale model of primary motor cortex circuits predicts in vivo cell-type-specific, behavioral state-dependent dynamics
Source: Cell Rep. Author manuscript; Available in PMC 2023 Oct 23. (PMC10592234; doi:10.1016/j.celrep.2023.112574)
Supplement: Supplementary Material [file NIHMS1922088-supplement-Supplementary_Material.pdf]

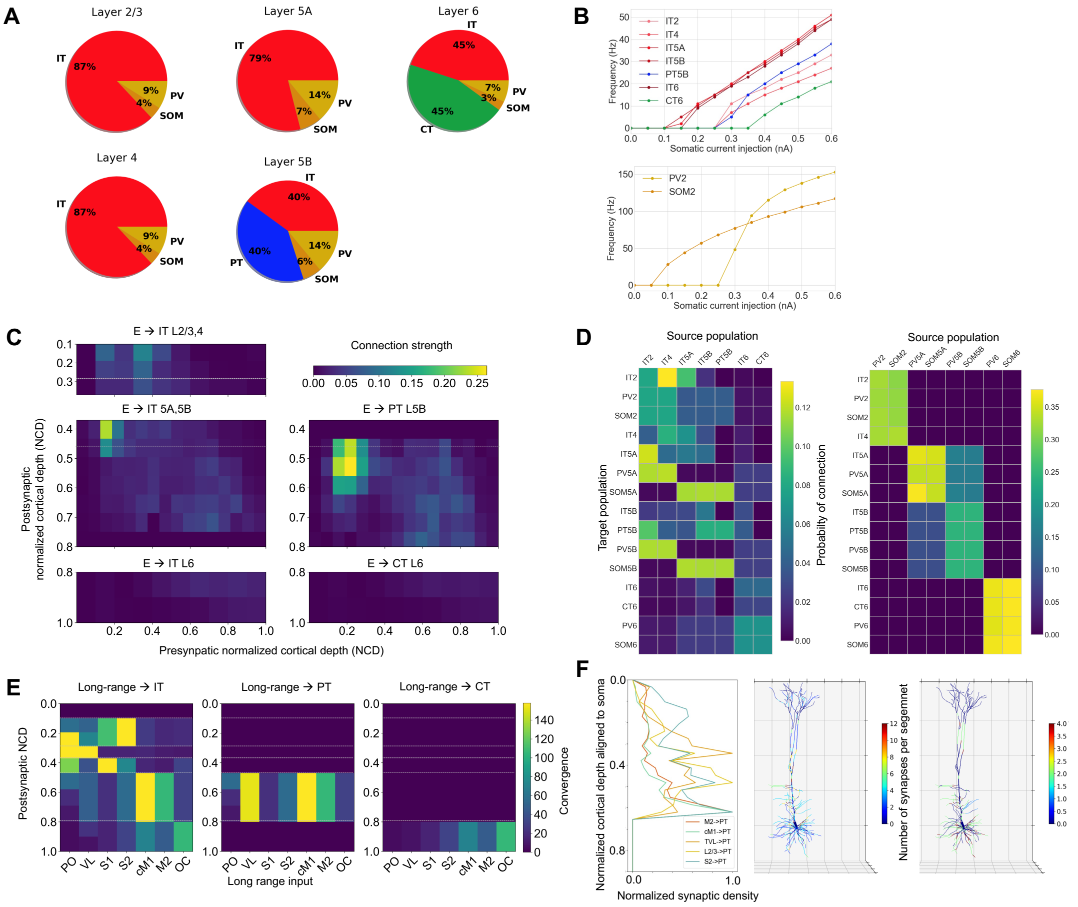

Figure S1. Microcircuit layer composition, cell type f-I response, local connectivity and long-range inputs. A. Proportion of cell classes per layer; **B.** f-I curve for each excitatory and inhibitory cell types. All properties were derived from published experimental data. Populations labels include the cell class and layer, e.g. 'IT2' represents the IT class neurons in layer 2/3. **C.** Strength of local excitatory connections as a function of pre- and post-synaptic normalized cortical depth (NCD) and post-synaptic cell class; values used to construct the network. **D.** Convergence of long-range excitatory inputs from seven thalamic and cortical regions as a function of post-synaptic NCD and cell class; values used to construct the network. **E.** Probability of connection matrix for excitatory (left) and inhibitory (right) populations calculated from an instantiation of the base model network. **F.** Left. Synaptic density profile (1D) along the dendritic arbor for inputs from layer 2/3 IT, VL, S1, S2, cM1 and M2 to PT neurons. Calculated by normalizing sCRACM maps (Ref[30] Figs. 5 and 6) by dendritic length at each grid location and averaging across rows. Middle and Right. Synaptic density per neuron segment automatically calculated for each neuron based on its morphology and the pre- and postsynaptic cell type-specific radial synaptic density function. Here, VL →PT and S2 →PT are compared and exhibit partially complementary distributions.

Experimental manipulation	Behavioral State	MTh input	NA input (PT I_{h})
Control	Quiet	Low (0-2.5 Hz)	Low NA (75% I_{h})
Control	Movement	High (0-10 Hz)	High NA (25% I_{h})
MTh inactivation	Quiet	Very low (0-0.01 Hz)	Low NA (75 % I_{h})
MTh inactivation	Movement	Very low (0-0.01 Hz)	High NA (25% I_{h})
NA-R block	Quiet	Low (0-2.5 Hz)	Very low (100% I_{h})
NA-R block	Movement	High (0-10 Hz)	Very low (100% I_{h})

Table S1. Motor thalamic (MTh) and noradrenergic (NA) input associated with the different experimental manipulations and behavioral states simulated in the M1 model. NA input is modeled by modifying the conductance of PT I_{h}.

**MTh inactivation condition**				
**Condition / State**	Control/Quiet	Control/Move	MTh/Quiet	MTh/Move
Control/Quiet	N/A	0.43	0.26	0.27
Control/Move	0.43	N/A	0.57	0.60
MTh/Quiet	0.26	0.56	N/A	0.14
MTh/Move	0.27	0.60	0.14	N/A
**NA block condition**				
**Condition / State**	Control/Quiet	Control/Move	NA/Quiet	NA/Move
Control/Quiet	N/A	0.60	0.20	0.30
Control/Move	0.60	N/A	0.68	0.52
NA/Quiet	0.20	0.68	N/A	0.47
NA/Move	0.30	0.52	0.47	N/A

Table S2. Silhouette Coefficients between the clusters of Fig. 5C,F.

MTh input	0.01 Hz	2.50 Hz	5.00 Hz	7.50 Hz	10.0 Hz
0.01 Hz	N/A	0.11	0.56	0.72	0.74
2.50 Hz	0.11	N/A	0.35	0.56	0.60
5.00 Hz	0.56	0.36	N/A	0.41	0.48
7.50 Hz	0.72	0.56	0.41	N/A	0.21
10.0 Hz	0.74	0.60	0.48	0.21	N/A

Table S3. Silhouette Coefficients between the clusters of Fig. 6 B (top panel).

NA input	0%	25%	50%	75%	100%
0%	N/A	0.27	0.47	0.70	0.73
25%	0.27	N/A	0.27	0.57	0.63
50%	0.47	0.27	N/A	0.45	0.60
75%	0.70	0.57	0.45	N/A	0.37
100%	0.73	0.63	0.60	0.37	N/A

Table S4. Silhouette Coefficients between the clusters of Fig. 6B (bottom panel)

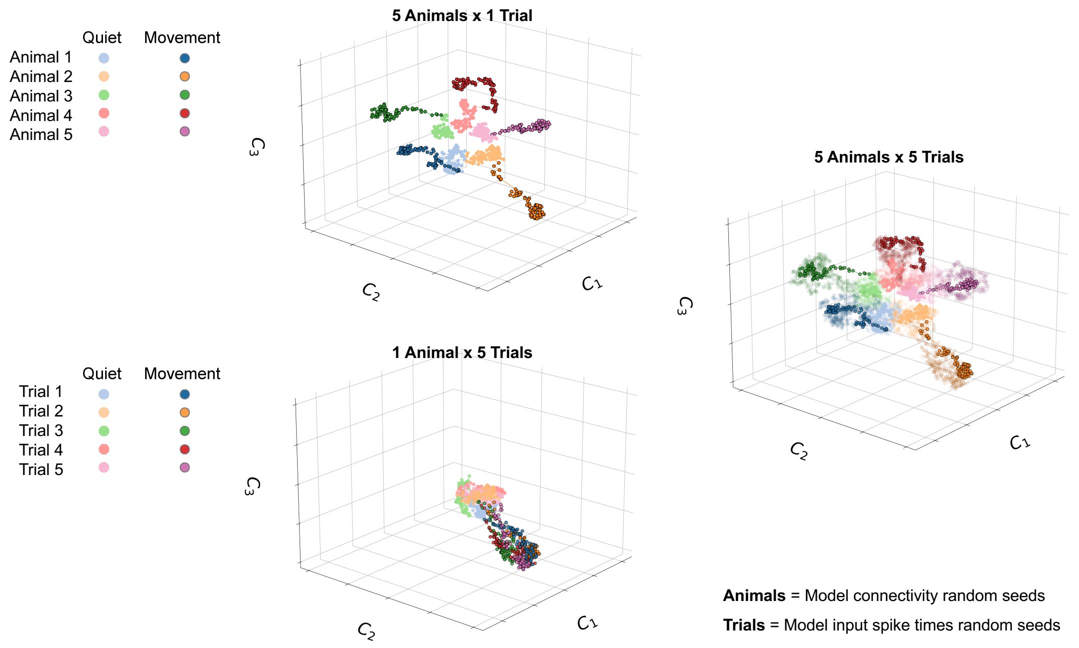

Figure S2. Low-dimensional representation (UMAP) of the firing rates of 1000 randomly selected neurons across different simulated animals and trials during the quiet and movement behaviors. Each point represents the firing rate within a 25 ms time step, for a duration of 2s for the quiet and 2s for the movement behavior. Different animals were simulated by modifying the model connectivity randomization seed, and different trials by modifying the model input spike times randomization seed. **Top-left.** Representation of 5 simulations corresponding to a single trial for 5 different animals (different colors) across quiet (light) and movement (dark). Shows separable data point clusters for each animal but similar quiet vs movement topological structures (manifolds) within each animal. **Bottom-left.** Representation of 5 simulations corresponding to 5 trials (different colors) for a single animal across quiet (light) and movement (dark). Shows a strong overlap of the data points for each trial but a separation of the quiet and movement conditions. **Right.** Representation of 25 simulations corresponding to 5 trials (4 shown semitransparent) across five animals (different colors). Illustrates the similarity of trials within each animal and the separability across animals.

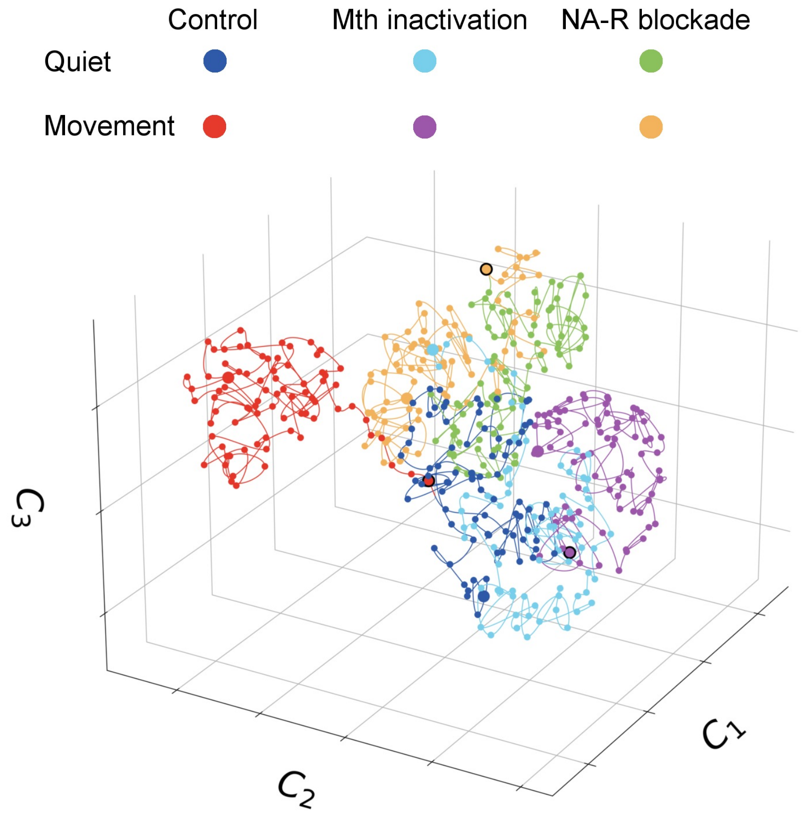

Figure S3. Low-dimensional representation (UMAP) of the firing rates of 1000 randomly selected neurons across different behaviors and conditions in the modified models. The control movement cluster (red) was markedly separable from the rest of clusters. The movement clusters of the MTh inactivation and NA-R block conditions (purple and orange) exhibit overlap with the quiet clusters (blue, cyan and green). The modified models include increased K+ conductance for the NA-R block condition, and decreased long-range inputs from both cM1 and M2 for the MTh inactivation condition. All behaviors and conditions are projected on the same low-dimensional space. Each point represents the firing rate within a 25 ms time step, for a duration of 2s for the quiet and 2s for the movement behavior. Start and end points indicated with larger dots; behavior transition point indicated with black border.
